# Effect of Encapsulated Phenolic Compounds of Cocoa on Growth of Lactic Acid Bacteria and Antioxidant Activity of Fortified Drinking Yogurt

**DOI:** 10.3390/molecules29143344

**Published:** 2024-07-16

**Authors:** Milena Rogalska, Joanna Oracz, Elżbieta Klewicka, Dorota Żyżelewicz

**Affiliations:** 1 Institute of Food Technology and Analysis, Faculty of Biotechnology and Food Sciences, Lodz University of Technology, 2/22 Stefanowskiego Street, 90-537 Lodz, Poland; milenak46@gmail.com (M.R.); joanna.oracz@p.lodz.pl (J.O.); 2 Institute of Fermentation Technology and Microbiology, Faculty of Biotechnology and Food Sciences, Lodz University of Technology, 171/173 Wólczańska Street, 90-530 Lodz, Poland; elzbieta.klewicka@p.lodz.pl

**Keywords:** cocoa, phenolic compounds, inclusion complexes, cyclodextrins, alginate–chitosan complexes, lactic acid bacteria

## Abstract

The aim of this study was to obtain drinking yogurts enriched with ACTICOA cocoa powder (ACTICOA), its extract (EACTICOA) and pure phenolics, as well as their inclusion complexes with cyclodextrins and alginate–chitosan (A-Ch) capsules, and to evaluate the effects of these additives on the viability of lactic acid bacteria (LAB) and antioxidant properties of fresh yogurts and yogurts stored for 14 days at 4 °C. The application of cocoa phenolic compounds in free form and in the form of EACTICOA to yogurts resulted in the greatest increase in the concentration of phenolic compounds and a significant improvement in the antioxidant properties of the fortified products. The highest TPC was found in yogurts enriched with free quercetin (107.98 mg CE/g). Yogurt fortified with free gallic acid showed the highest ability to neutralize free radicals (EC50 = 2.74 mg/mg DPPH, EC50 = 5.40 mg/mg ABTS) and reduce ferric ions (183.48 µM Trolox/g). The enrichment of yogurts with the tested phenolic compounds preparations, especially in the form of encapsulates, did not affect the viability of LAB during storage.

## 1. Introduction

Yogurt is a traditional fermented milk product obtained by fermenting milk with lactic acid bacteria—*Streptococcus thermophilus* and *Lactobacillus delbrueckii* subsp. *bulgaricus* [1]. It is classified as a product with health-promoting properties, low in fat and high in protein. Yogurt is also a source of microelements (magnesium compounds), macroelements (phosphorus, potassium, calcium compounds) and B vitamins [2]. On average, yogurt contains approximately 10^8^ cfu/g of lactic acid bacteria cells belonging to the mentioned species. Although the number of these bacteria decreases significantly during passage through the human digestive tract (10^4^–10^6^ cfu/g of feces), they can positively interact with the human commensal microbiota [3]. Epidemiological data show that consuming yogurt among adults has health benefits. For example, yogurt consumption was associated with a 28% reduction in the incidence of type 2 diabetes [4]. Moreover, it was found that consuming yogurt contributes to the improvement of the metabolic profile, lowers the level of free triglycerides and glucose, lowers blood pressure and reduces the resistance of cells to insulin [5]. Additionally, yogurt and other fermented dairy products have a positive effect on the human digestive tract, mainly by stabilizing and regulating the intestinal microbiota [6]. Moreover, in recent years, there have been reports on the preventive role of yogurt and other milk-based fermented drinks against common respiratory infections in both adults and children [7,8].

From the point of view of the growing functional food segment, yogurt is an excellent base for this type of product. Classic yogurt can be enriched with probiotic bacteria, fiber, or bioactive compounds of plant origin, e.g., polyphenols. The literature provides examples of the introduction of phenolic compounds from olives [9], apples [10], tea, purple potato [11] and berry fruits, including blackcurrant pomace, pomegranate peel and grape extract [11,12,13,14], into yogurts. However, phenolic compounds used for food supplementation, including yogurts, may lose their health-promoting properties due to their instability over time and under various environmental conditions (temperature, oxygen, pH, enzymatic activity of bacteria). This is confirmed by the research of Ho et al. [15,16], which showed that (+)-catechin present in the inclusion complex with cyclodextrins was characterized by better antioxidant properties than in the free form. In this case, β-cyclodextrin (β-CD) and its derivative had a protective effect on (+)-catechin. The obtained complex of (+)-catechin with cyclodextrins was introduced into dairy products such as cheese, milk and traditional yogurt. A study conducted by the same research group showed that this complex was more stable in milk and traditional yogurt than in cheese. Caffeic, chlorogenic and quinic acids were also used to form inclusion complexes with β-CD and hydroxylpropyl-β-CD (HP-β-CD). It has been shown that cyclodextrins increase the bioactive properties of encapsulated substances [17,18]. He et al. [16] used β-CD and its derivative to encapsulate oxyresveratrol, which increased the stability of this substance in fresh grape juice. The effectiveness of encapsulation with β-CD and HP-β-CD in terms of increasing the stability of encapsulated phenolic compounds was also observed in the case of peach pomace [19], red grape pomace [20] and raw Robusta coffee beans [21]. There is also research available on the use of biopolymers such as alginate and chitosan to create spherical structures with a bioactive interior. These compounds and the encapsulates obtained from them are non-toxic and biodegradable [22,23]; therefore, they are used in the food industry in the production of e.g., sauces, ice cream, or fruit cakes to thicken or stabilize them [24]. Alginate additionally has antioxidant properties and is stable in the acidic pH of the stomach. Only in the alkaline environment of the small intestine does it gradually release, which has a beneficial effect on its use in food or dietary supplements [25,26,27]. Sodium alginate combined with chitosan was used to encapsulate lactic acid bacteria [28], bovine serum albumin [29], Yerba mate tea [30] and anthocyanins extracted from mulberries [31] and black rice grains [32].

The aim of the research was to obtain drinking yogurts enriched with various preparations of phenolic compounds in the form of (a) ACTICOA cocoa powder (ACTICOA) (Barry Callebaut Poland Ltd., Łódź, Poland), (b) ACTICOA extract (EACTICOA), (c) pure phenolic compounds ((+)-catechin (+)-C, gallic acid (GA) and quercetin (Q)), (d) phenolic compounds inclusion complexes with β-CD and its derivative HP-β-CD obtained by the classic method, (e) ACTICOA inclusion complexes with β-CD and its derivative HP-β-CD obtained as a result of reactive extraction and (f) alginate–chitosan capsules (A-Ch) with EACTICOA or (+)-catechin obtained using two methods (v.1, v.2). Next, an assessment of the impact of the mentioned preparations on the antioxidant and anti-free-radical activity (in vitro) of the prepared yogurts was carried out. Moreover, the influence of the bioactive ingredients used on the number of lactic acid bacteria in fresh yogurts and yogurts stored for 14 days in refrigerated conditions was assessed.

## 2. Results and Discussion

### 2.1. Characteristics of Cocoa Phenolic Compounds Preparations

Chromatographic analysis showed that ACTICOA and EACTICOA contained mainly phenolic compounds from the flavonoid group, including (+)-C, (−)-epicatechin and procyanidins B2 and C1 (Table 1). Figures S1–S21 show typical UHPLC DAD chromatograms of ACTICOA, EACTICOA, pure phenolic compounds and their encapsulates. The results of the identification of the phenolic compounds using the UHPLC-DAD-ESI-MS/MS method are summarized in Table S1. Phenolic acids such as GA, protocatechuic acid, caffeic acid aspartate and *p*-coumaric acid aspartate were also found in both the cocoa powder and the extract obtained from it. Among these phenolic compounds, (−)-epicatechin was predominant in both preparations. It was also found that EACTICOA contains five times more flavonoids, phenolic acids and their derivatives than the cocoa powder from which it was obtained.

The formation efficiency, composition and concentration of phenolic compounds and total content of phenolic compounds (TPC) of the tested encapsulates are presented in Table 2 and Table 3. It was observed that the process of forming (+)-C inclusion complexes with HP-β-CD was more effective than complexing this compound with β-CD. In the case of (+)-C-HP-β-CD, catechin was almost completely complexed, and the efficiency of complex formation was 98.90% (Table 2). The efficiency of complexation of (+)-C with β-CD was approximately 1/3 lower and was determined to be 67.81%. Similar results were obtained for the complexation efficiency of GA with both β-CD and HP-β-CD. Conversely, the complexation efficiencies of Q using β-CD and HP-β-CD were much lower, reaching 55.75% and 62.46%, respectively. The obtained results are similar to previously published research data, in which the complexation efficiency depended on the temperature and process time and ranged from 61.5 to 64.9% [33]. Other studies also showed an increase in complexation efficiency using HP-β-CD solution [34]. The benefits of using CDs for extraction have also been observed in the case of the extraction of phenolic compounds from onion waste [35], bush sage [36], or olive leaves [37]. The applied process of obtaining EACTICOA and (+)-C, Q and GA alginate–chitosan capsules using two methods allowed for almost complete complexation of the introduced phenolic compounds. The encapsulation efficiency for these capsules ranged from 94.78 to 99.49% (Table 3). Similar encapsulation efficiencies of phenolic compounds in A-Ch complexes were observed by Carrasco-Sandoval et al. [38].

The studies showed that the tested complexes were characterized by different TPC values. In the case of (+)-C complexes with CDs, a higher content of this compound was determined in the complexes with β-CD, which amounted to 202.26 mg CE/g (Table 2). In the inclusion complex (+)-C with HP-β-CD, the TPC was 165.03 mg CE/g. Similar relationships were found for GA inclusion complexes with CDs. In contrast, Q, complexes with HP-β-CD were characterized by a higher TPC than in the β-CD complex (294.89 and 211.53 mg CE/g). A similar relationship was observed in the studies of Abril-Sánchez et al. [39].

A higher TPC value was also determined for the ACTICOA-β-CD(RE) inclusion complex than for ACTICOA-HP-β-CD(RE). The results of TPC determination in A-Ch-(+)-C, A-Ch-Q, A-Ch-GA and A-Ch-EACTICOA capsules obtained by two different methods showed that capsules containing Q as the active core had a higher value of this parameter. However, TPC varied among capsules containing the same active core but obtained by a different method (Table 3). The capsules with Q, (+)-C, GA and EACTICOA obtained in version 1 were characterized by a much higher TPC, amounting to 189.63, 73.50, 69.40 and 32.46 mg CE/g, respectively, than the preparations obtained in version 2. The antioxidant activity of the tested preparations was determined using three in vitro methods, i.e., the ability to scavenge DPPH or ABTS free radicals and the ability to reduce iron ions (Table 4). It was found that inclusion complexes with CDs have different abilities to neutralize DPPH and ABTS free radicals. In comparison with ACTICOA, EACTICOA and free phenolic compounds, their inclusion complexes with CDs showed a lower ability to neutralize the above-mentioned free radicals. In the DPPH radical test, the EC50 values for ACTICOA, EACTICOA and free (+)-C, Q, and GA were 0.55, 0.20, 0.23, 0.21 and 0.15 mg/mg DPPH, respectively, while for their complexes with CD ranges from 1.56 mg/mg DPPH for GA-β-CD to 4.01 mg/mg DPPH for (+)-C-HP-β-CD complex. 

Similarly, in the ABTS test, the highest antioxidant capacity was observed for free (+)-C (EC50 = 0.05 mg/mg ABTS), and the lowest for ACTICOA-HP-β-CD(RE) (EC50 = 1.45 mg/mg ABTS). It was also found that β-CD complexes had greater anti-free-radical activity than HP-β-CD complexes in the DPPH test. An opposite behavior was observed for the ability to neutralize free cationic ABTS radicals. The ability of inclusion complexes with β-CD and HP-β-CD to scavenge the above-mentioned free radicals was also confirmed in the studies conducted by Zhang et al. [40]. The free radical scavenging capacity in the ABTS test of A-Ch-(+)-C, A-Ch-Q, A-Ch-GA and A-Ch-EACTICOA capsules obtained by the two different methods was clearly different. It was observed that capsules containing Q as the active core had the highest antioxidant activity (EC50 = 1.87 mg/mg ABTS), while those obtained with EACTICOA had the lowest (EC50 = 9.89 mg/mg ABTS). It was also shown that all the formulations obtained had a significantly higher ability to neutralize ABTS cation radicals than DPPH free radicals. In the case of determining the ability to reduce iron(III) ions in the FRAP test, relationships similar to the results of the TPC analysis and the ability to reduce ABTS cation radicals were observed. The (+)-C, Q, GA and ACTICOA complexes with HP-β-CD had higher antioxidant activity in the FRAP test than the preparation with β-CD. Complexes with HP-β-CD were characterized by a greater power to reduce these ions than analogous preparations obtained with β-CD. This may be due to the larger size of the HP-β-CD cavity than that of β-CD, which translates into more effective complexation of bioactive substances and greater protection of the health-promoting properties of the preparations [40]. It was observed that the antioxidant activity of pure phenolic compounds and ACTICOA in the form of inclusion complexes with CDs and in the form of A-Ch capsules was lower than that of its free form. The obtained results are consistent with literature data regarding the properties of curcumin complexes with β-CD, γ-CD and HP-β-CD. They indicate that the inclusion of curcumin in the cyclodextrin molecule increases its stability, but the antioxidant activity of this compound is reduced. Presumably, as a result of the formation of inclusion complexes of phenolic compounds, the active functional groups responsible for their anti-free-radical activity, reducing power and ability to chelate heavy metal ions may be blocked [41]. Among the A-Ch capsules, the preparation A-Ch-(+)-C v.1 (729.42 µM Trolox/g) had the best ability to reduce iron(III) ions. However, the weakest properties were found for A-Ch-EACTICOA v.2 capsules (180.75 µM Trolox/g). Method 1 of obtaining capsules in the alginate–chitosan system, with a shorter shaking time (v.1), had an impact on increasing the reducing capacity of the tested preparations. The A-Ch-EACTICOA v.1 preparation was characterized by a higher reducing power in the FRAP test (200.52 µM Trolox/g) than analogous capsules, but obtained by method 2 (v.2), similarly to the case of A-Ch capsules with (+)-C and GA whose higher iron ion-reducing abilities were determined in preparations obtained using method 1 (the exception was the Q capsules). The iron(III) ion-reducing properties of A-Ch capsules, determined in the FRAP test, were confirmed in the professional literature, e.g., in the studies of Haładyn et al. [42], who analyzed the above-mentioned properties of A-Ch capsules with the addition of guar gum. 

### 2.2. Number of Lactic Acid Bacteria in Yogurts

The results of the microbiological analysis presented in Table 5 are expressed as log (cfu/mL) SD (LU). The obtained yogurt contained *Lactobacillus* spp. bacteria at the level of 7.7 ± 0.43 LU. The number of *Streptococcus* spp. bacteria was 9.3 ± 0.07 LU. After storing the yogurt at 4 °C for 14 days, the bacterial count was 6.5 ± 0.76 LU and 8.8 ± 0.23 LU, respectively. For both groups of bacteria, the reduction in abundance was statistically significant.

Yogurt with the addition of Q, GA, inclusion complexes β-CDs with these phenolic compounds, preparations with CDs obtained as a result of reactive extraction, A-Ch capsules with the addition of selected cocoa phenolic compounds obtained by method 2 and with Q and GA obtained by method 1 after 2-week storage are characterized by no statistically significant differences (in the case of *Lactobacillus* spp. bacteria) versus yogurt after fermentation. No statistically significant differences (in the case of *Streptococcus* spp. bacteria) were observed between the yogurt after fermentation and yogurt with the (+)-C-β-CD and the inclusion complex with HP-β-CD, obtained as a result of reactive extraction and A-Ch capsules with each tested phenolic compounds in both methods used. Statistically significant differences (in the case of *Streptococcus* spp. bacteria) were found between pure yogurt after storage for 14 days, and yogurt enriched with ACTICOA, (+)-C, GA, (+)-C-β-CD, Q-HP-β-CD and ACTICOA-β-CD(RE) preparations.

GA significantly reduced the number of *Lactobacillus* spp. bacteria. The number of these bacteria after 14 days of storage in yogurt without additives was 6.5 ± 0.76 LU, while in the case of yogurt with GA, it was 3.5 ± 1.33 LU. A similar phenomenon was observed for *Streptococcus* spp. bacteria. The number of these bacteria was 8.8 ± 0.23 LU and 5.2 ± 0.00 LU, respectively. The gallic acid encapsulation process significantly improved the survival of bacteria compared to samples without gallic acid encapsulation after storage. For the preparations GA-β-CD inclusion complex, GA-HP-β-CD inclusion complex and capsules A-Ch-GA v.1 and v.2, the number of *Lactobacillus* spp. bacteria increased accordingly by 3.4 LU, 2.1 LU, 2.2 LU and 3.3 LU, while for *Streptococcus* the following increases were recorded: 2.9 LU, 2.6 LU, 3.3 LU and 1.5 LU, respectively. Based on the results obtained, we can conclude that the gallic acid encapsulation process protects lactic acid bacteria present in yogurt against the unfavorable antagonistic effects of this compound. Low concentrations of gallic acid significantly stimulate the growth and metabolic activity of *Lactobacillus* bacteria. In the case of high concentrations of gallic acid (above 3 mM), the integrity of the bacterial cell walls is disrupted, resulting in a disturbed pH gradient [43,44].

Similar results were obtained by Jakubowska et al. [45]. In the natural yogurts tested by them, a slight decrease in the amount of yogurt microorganisms occurred during 3-week storage in refrigerated conditions. Conversely, in the studies by Romero et al. [46] the addition of green tea phenolic compounds to yogurt did not affect the viability of lactic acid bacteria during their storage.

It is known that Gram-positive bacteria are much more susceptible to the unfavorable (antagonistic) effects of phenolic compounds [47]. Therefore, the introduction of food rich in microorganisms of phenolic compounds and their derivatives into the environment should take into account the sensitivity of these microorganisms. When using biologically active compounds, the concentration and form of introduction into food, in this case into yogurt, are important. Due to the metabolic abilities of lactic acid bacteria, attention should also be paid to the possibility of degradation of the introduced bioactive compounds [44].

The study conducted by Feng et al. [48] showed that encapsulation of tea phenolic compounds preparations introduced in this form into yogurt protected bioactive compounds by 91.58%. Moreover, tea phenolic compounds introduced into the milk environment did not disturb the yogurt fermentation processes. These results are consistent with those that we obtained.

According to the requirements set out in the FAO/WHO Codex Alimentarius standard, the number of characteristic microflora in yogurt must be at least 10^7^ cfu in 1 g or mL of the product throughout the shelf life of the yogurt [49]. Therefore, the yogurts we tested with the majority of the preparations of phenolic compounds meet the criteria for yogurts.

### 2.3. Total Phenolic Content

**Figure 1:**
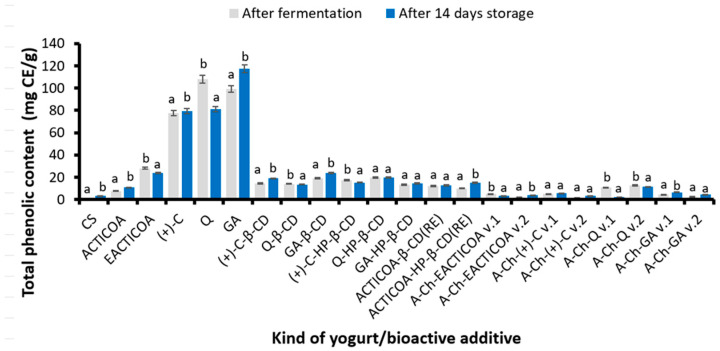
Total phenolic content in yogurts enriched with various preparations of cocoa phenolic compounds after their production and after 14 days of refrigerated storage. Values are presented as mean ± SD (*n* = 6). Bars that share the same superscript letter (a,b) are not significantly different from each other (Tukey’s HSD test, *p* < 0.05). Abbreviations of tested samples as in Table 4.

Figure 1 shows the content of total phenolic compounds (TPC) in yogurt without additives, immediately after the fermentation process and in yogurts enriched with the addition of ACTICOA, EACTICOA, phenolic compounds present in cocoa and cocoa products ((+-C), Q, GA), inclusion complexes (+)-C, Q, GA with cyclodextrins (β-CD, HP-β-CD) obtained by the classical method andACTICOA inclusion complexes with the above-mentioned cyclodextrins obtained as a result of reactive extraction or A-Ch capsules with (+)-C, Q or GA obtained by two methods (v.1 or v.2) after production (fermentation and addition of phenolic compounds preparations) and 14 days of refrigerated storage.

It was observed that yogurt after fermentation without additives was characterized by the lowest content of phenolic compounds (1.23 mg CE/g DW). The presence of phenolic compounds in natural yogurt may be related to the fact that the Folin–Ciocalteu method is not specific only to phenolic compounds. Other compounds with reducing properties (reducing sugars, amino acids, peptides) may also react with the Folin–Ciocalteu reagent and interfere with the assay results [50]. Therefore, this method can be used to determine not only phenolic compounds but also other substances with reducing power. An example of an amino acid that could have an impact on the increase in TPC content is tyrosine, which has a phenolic side chain [51].

Directly after production, the addition of the obtained various types of preparations of phenolic compound increased TPC in the tested yogurts. As expected, the highest TPC was recorded in yogurts enriched with free Q (107.98 mg CE/g), GA (99.09 mg CE/g), or (+)-C (77.49 mg CE/g). EACTICOA fortified yogurt had a much lower, but still relatively high, TPC (28.21 mg CE/g). The tested dairy products were also enriched with the addition of inclusion complexes with cyclodextrins. It was found that yogurts enriched with inclusion complexes of free compounds with CDs had a higher TPC than preparations of ACTICOA complexes obtained by reactive extraction. It was also shown that, with the exception of yogurts with GA inclusion complexes with CDs, yogurts with preparations obtained with HP-β-CD had a higher TPC than analogous products but with complexes with β-CD. Yogurts enriched with A-Ch capsules were characterized by a much lower TPC than in the case of control yogurts (immediately after production, without additives) and those fortified with free (+)-C, Q, GA, or cyclodextrin inclusion complexes. Yogurts with the addition of A-Ch capsules obtained by method 1 had a higher content of these compounds than analogous products enriched with capsules produced by method 2. The lowest TPC was determined in yogurt enriched with capsules with (+)-C obtained by method 2 (1.71 mg CE/g). Similar conclusions were observed in the case of enriching yogurts with green tea extract [52] or grape extract [12].

Based on the results of storage tests, it was shown that during 14 days of storage of yogurt with the addition of GA or (+)-C, the TPC increased by approximately 18.5 and approximately 2.5%, respectively. Yogurt with ACTICOA and most yogurts enriched with the tested encapsulates were also characterized by a higher TPC value after storage, except for products with EACTICOA, Q-β-CD, (+)-C-HP-β-CD, A-Ch-EACTICOA v.1, A-Ch-Q v.1 and A-Ch-Q v.2, in which a decrease in TPC was observed.

Research conducted by other scientists shows that storing fortified yogurts does not reduce the concentration of phenolic compounds in these products [14]. For example, yogurts supplemented with dried berries and incubated at 4 °C for 4 weeks were characterized by a constant content of phenolic substances [14]. Moreover, in studies on yogurts with the addition of lotus leaves rich in phenolic compounds, an increase in the concentration of these bioactive substances was observed during their refrigerated storage for 14 days [53].

### 2.4. Antioxidant Activity

The antioxidant activity of yogurts was examined using in vitro tests as the ability to scavenge DPPH free radicals, ABTS cation radicals, and ferric reducing ability (FRAP).

#### 2.4.1. Free Radical Scavenging Capacity (DPPH)

**Figure 2:**
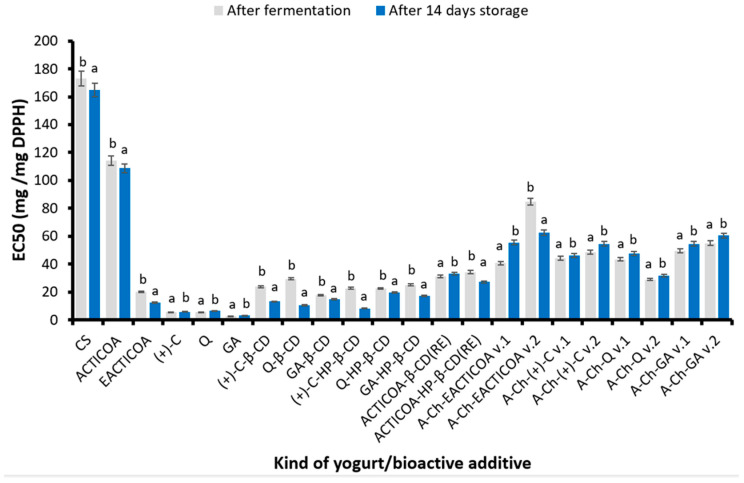
Free radical scavenging capacity (DPPH) of yogurts enriched with various preparations of cocoa phenolic compounds after their production and after 14 days of refrigerated storage. Values are presented as mean ± SD (*n* = 6). Bars that share the same superscript letter (a,b) are not significantly different from each other (Tukey’s HSD test, *p* < 0.05). Abbreviations of tested samples as in Table 4.

The DPPH test results are presented in Figure 2 as the EC50 parameter in mg of product per mg of DPPH radical.

It was observed that yogurt, both without additives and supplemented with ACTICOA, had only a slight ability to scavenge DPPH free radicals. Such negligible free radical neutralizing properties in this case may result from the interactions of milk proteins with phenolic compounds present in cocoa powder and the degradation of these substances [42]. The highest inhibition capacity of the above-mentioned radical among enriched yogurts was characterized by the product with added free GA (EC50 = 2.74 mg/mg DPPH). 

In the case of yogurts with the addition of inclusion complexes, it was found that the best ability to neutralize DPPH radicals was demonstrated by yogurts enriched with GA complexes with β-CD and HP-β-CD, while the product supplemented with GA-β-CD had a higher ability to inhibit DPPH radicals (EC50 = 17.50 mg/mg DPPH). The positive effect of inclusion complexes on the scavenging capacity of DPPH free radicals has also been described in the studies of other researchers [19,54,55,56,57].

Analyzing the results of tests on the ability to scavenge DPPH free radicals by yogurts enriched with A-Ch capsules of cocoa phenolic compounds, it should be concluded that better results in this respect were obtained in the case of yogurts with encapsulates obtained using method 1. Despite the fact that in most cases, yogurts with these capsules obtained using method 1 were characterized by a greater ability to neutralize DPPH radicals, among all products with A-Ch capsules, yogurt with the addition of A-Ch-Q v.2 had the highest ability to scavenge these radicals (EC50 = 28.91 mg/mg DPPH). The lowest value of the discussed parameter was recorded in yogurt with the addition of A-Ch-EACTICOA v.2 (EC50 = 84.75 mg/mg DPPH).

It was shown that two weeks of refrigerated storage of yogurts resulted in an increase in the antioxidant properties of most products in the DPPH test. Most yogurts enriched with ACTICOA, EACTICOA and inclusion complexes showed high stability during 14 days of refrigerated storage. The exceptions were dairy products with the addition of ACTICOA-β-CD(RE) or (+)-C, as well as most yogurts with A-Ch capsules (except the product with A-Ch-EACTICOA v.2—37% increase in free radical neutralizing properties), in which there was a decrease in the ability to scavenge the above-mentioned radicals.

The conducted research showed that yogurts enriched with the obtained preparations of phenolic compounds were characterized by increased antioxidant activity in the DPPH test compared to the control sample (yogurt without additives). It was observed that the use of most of the obtained preparations for yogurt fortification also contributed to the increase in the ability to scavenge DPPH free radicals during 14 days of storage of this product, except for the majority of A-Ch encapsulates. The obtained results confirmed that antioxidants contained in the preparations obtained from ACTICOA and selected cocoa phenolic compounds retain their anti-free-radical activity even during storage [34]. Similar results were obtained by Kim et al. [53] in the case of supplementing yogurts with lotus leaf powder rich in phenolic compounds. These products had a greater ability to scavenge DPPH radicals than natural yogurt without this additive [53].

#### 2.4.2. ABTS Cation Radical Scavenging Ability

**Figure 3:**
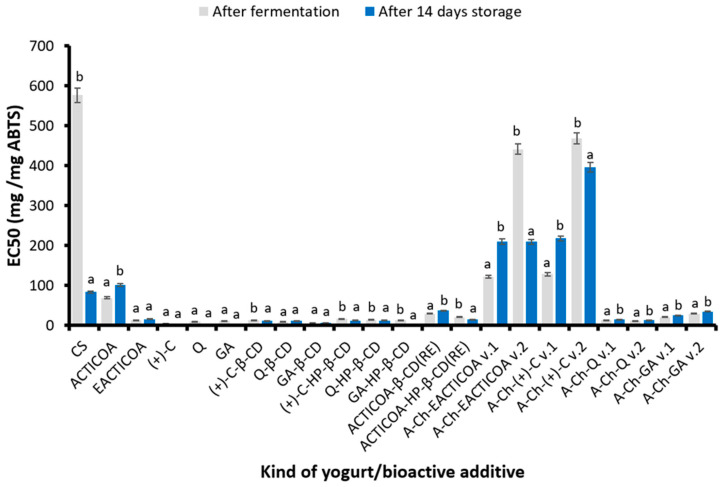
ABTS cation radical scavenging ability of yogurts enriched with various preparations of cocoa phenolic compounds after their production and after 14 days of refrigerated storage. Values are presented as mean ± SD (*n* = 6). Bars that share the same superscript letter (a,b) are not significantly different from each other (Tukey’s HSD test, *p* < 0.05). Abbreviations of tested samples as in Table 4.

The results of determining the ability of the obtained yogurts to scavenge free ABTS cation radicals are presented in Figure 3.

Similarly to the anti-free-radical activity in the DPPH test, enriching yogurts with the obtained preparations of cocoa phenolic compounds contributed to a significant increase in their ability to scavenge ABTS cation radicals. The best anti-free-radical activity in the ABTS test was demonstrated by yogurt fortified with free GA (EC50 = 5.40 mg/mg ABTS), followed by yogurts fortified with free Q, GA, or (+)-C inclusion complexes with β-CD and GA or Q inclusion complexes with HP-β-CD. Yogurts enriched with other preparations of phenolic compounds obtained as part of the research had weaker anti-free-radical activity in the ABTS test than the above-mentioned ones. Yogurts with the addition of A-Ch capsules obtained by method 1 had a higher ability to scavenge ABTS radicals than analogous capsules obtained by method 2. A shorter shaking time in method 1 resulted in better anti-radical properties. Moreover, it was found that yogurts enriched with A-Ch capsules with Q or GA had a higher ability to scavenge the ABTS cation radical than products with A-Ch capsules with EACTICOA and (+)-C.

During two weeks of refrigerated storage of yogurts, an increase in the ability to scavenge the ABTS radical was observed both in the product without the addition of obtained preparations and in yogurts enriched with the following preparations: free phenolic compounds—(+)-C, GA or Q, inclusion complexes of cocoa phenolic compounds with both cyclodextrins, which were prepared using the classical method, ACTICOA-HP-β-CD(RE), A-Ch-EACTICOA v.2 and A-Ch-GA v.1.

An increase in the ability to scavenge the above-mentioned radicals in the tested products was also observed when enriching yogurts with the addition of various types of honey [58], green tea rich in phenolic compounds [59], grape seeds [60], black carrot concentrate [61], chokeberry juice [62], or herbal extracts and spices [63,64,65].

#### 2.4.3. Ferric Reducing Ability (FRAP)

**Figure 4:**
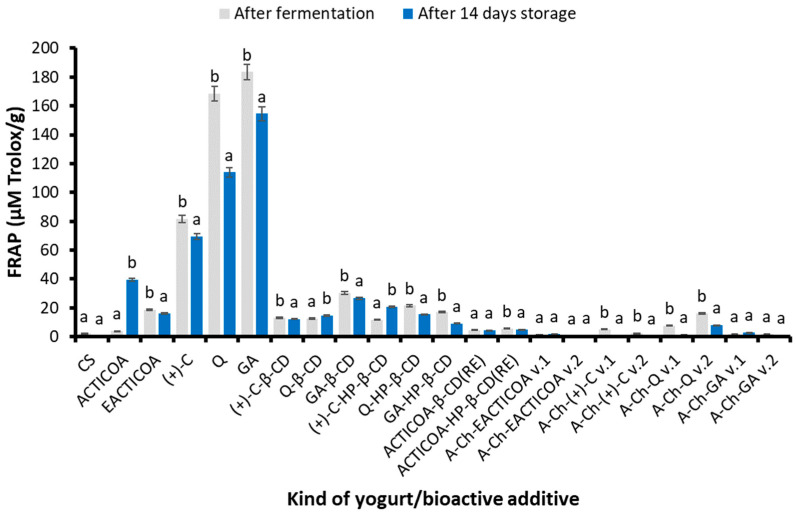
Ferric reducing ability of yogurts enriched with various preparations of cocoa phenolic compounds after their production and after 14 days of refrigerated storage. Values are presented as mean ± SD (*n* = 6). Bars that share the same superscript letter (a,b) are not significantly different from each other (Tukey’s HSD test, *p* < 0.05). Abbreviations of tested samples as in Table 4.

The research results of the FRAP test of the examined yogurts are presented in Figure 4. Yogurt with free GA (183.48 µM Trolox/g) and then with the addition of free Q (168.49 µM Trolox/g) demonstrated the best reducing properties in this test. Yogurt with (+)-C (81.51 µM Trolox/g) had a much lower, but still high, ability to reduce iron(III) ions. The product with EACTICOA was characterized by an over 4.5 times better iron(III) ion reducing capacity (18.51 µM Trolox/g) compared to the yogurt with ACTICOA (3.93 µM Trolox/g). High activity in this respect was also demonstrated by yogurt enriched with inclusion complexes obtained using the classical method, such as complex GA with β-CD (30.29 µM Trolox/g) and Q with β-CD (21.45 µM Trolox/g). 

In yogurts with inclusion complexes obtained as a result of reactive extraction, lower reduction values were obtained in the FRAP test than in the case of inclusion complexes obtained using the classical method but higher values than in the case of yogurt with ACTICOA. The beneficial effect of cyclodextrins (β-CD, HP-β-CD) on the reducing properties of iron(III) ions is also confirmed by other studies [34,66]. Yogurts with the addition of A-Ch capsules generally showed significantly lower reducing properties in the FRAP test than other examined fortified dairy products. The exception were yogurts with A-Ch-Q v.2 (16.1 µM Trolox/g) and A-Ch-(+)-C v.1 (5.19 µM Trolox/g). Among the enriched products, yogurt with A-Ch-EACTICOA v.2 capsules (0.64 µM Trolox/g) had the lowest ability to reduce iron(III) ions.

Storing pure yogurt and most fortified yogurts in refrigerated conditions for 2 weeks resulted in a decrease in reducing capacity in the FRAP test. A clear reduction in the reducing properties in the FRAP determination in the examined products after 5–6 h of storage was also confirmed in the research conducted by other teams. The reason for this phenomenon is the increased interaction of milk proteins with phenolic compounds [52,53,63,67]. Yogurt fortified with uncomplexed cocoa powder (ACTICOA) and yogurts enriched with Q-β-CD, (+)-C-HP-β-CD, A-Ch-EACTICOA v.1 and v.2 and A-Ch-GA v.1 behaved differently during refrigerated storage. These products showed higher iron(III) ion reducing power after storage. Presumably, this is due to the activity of microorganisms, which resulted in an increase in the content of compounds with the ability to reduce iron(III) ions. An increase in the antioxidant potential in the FRAP test in enriched yogurts compared to the control sample (without additives) was also found in studies described in the literature on yogurts with the addition of polyphenolic extracts from strawberry pomace or yogurts enriched with lotus leaves extract [53].

## 3. Materials and Methods

### 3.1. Materials

#### Chemical Reagents

The following chemicals and reagents were used in the research: hexane, ethanol (96%), Folin–Ciocalteu reagent (F-C), anhydrous sodium carbonate, methanol and potassium persulfate from Chempur (analytical grade; Piekary Śląskie, Poland), glacial acetic acid and hydrochloric acid (36%) from POCH S.A (analytical grade; Gliwice, Poland), β-cyclodextrin (β-CD), hydroxypropyl-β-cyclodextrin (HP-β-CD), sodium alginate, calcium chloride and chitosan from Sigma-Aldrich (Poznań, Poland), gallic acid, quercetin, (+)-catechin, 2,2-diphenyl-1-picrylhydrazyl (DPPH), 2,4,6-tri(2-pyridyl)-striazine (TPTZ), ferric chloride hexahydrate and 2,2′-azino-bis(3-ethylbenzothiazoline-6-sulfonic acid) diammonium salt (ABTS) from Sigma Aldrich (St. Louis, MO, USA) and phosphate buffer pH 3.6 from Fluka, Sigma-Aldrich (Schaffhausen, Switzerland). Deionized water was produced using the water purification system Direct-Q (Millipore Corp., Bedford, MA, USA). 

### 3.2. Preparation of Cocoa Phenolic Compounds Encapsulates

#### 3.2.1. ACTICOA Extract Preparation

EACTICOA was obtained according to the modified procedure described by Żyżelewicz et al. [68]. First, ACTICOA was defatted twice using n-hexane with the cocoa-powder-to-solvent ratio of 1:10 (*w*/*v*). Then, the defatted cocoa powder was extracted twice as follows. A total of 0.5 g of defatted ACTICOA was weighed into a Falcon centrifuge tube with a capacity of 15 mL and 5 mL of 70% ethanol solution was added and shaken for 10 min at 60 °C at the speed of 150 rpm/min using an SV 1422 Memmert (Schwabach, Germany) water bath shaker. Then, the sample was centrifuged using the Centurion Scientific K3 series centrifuge model K2015R (Stoughton, UK). Centrifugation parameters: 4800× *g*, temp. 4 °C, 20 min. The obtained supernatant was poured into a clean 15 mL centrifuge tube. The residue was poured back into 5 mL of 70% ethanol and shaken at ambient temperature at 150 rpm for 30 min using a Lab Companion OS-4000 orbital shaker (JEIO TECH, Yuseong-gu, Daejeon, Republic of Korea). Next, the sample was centrifuged at 4800× *g* at 4 °C for 20 min. After extraction, the obtained extracts (supernatants) were combined and filtered under reduced pressure through a paper filter with a pore diameter of 1.2 µm. Ethanol was evaporated from the filtrate using a rotary evaporator (Heidolph Instruments GmbH & Co. KG, Schwabach, Germany). The aqueous residue (water extract) was filtered under reduced pressure, frozen (−80 °C, 24 h) and freeze-dried (−50 °C, 0.9 mbar) using a DELTA 1-24 LSC Christ freeze dryer (Martin Christ, Osterode am Harz, Germany).

#### 3.2.2. Synthesis of Inclusion Complexes of Phenolic Compounds with β-CD or HP-β-CD Using Classical Method

The procedure for creating inclusion complexes of cocoa phenolic compounds ((+)-C, Q, GA) with cyclodextrins (β-CD, HP-β-CD) was carried out according to the modified method presented in the research of Żyżelewicz et al. [33] as follows. The tested substances were weighed in the 1:1 molar ratio of phenolic compound/β-CD or HP-β-CD. Then, 20 mL of deionized water was added and the whole mixture was shaken in an SV 1422 Memmert water bath (Schwabach, Germany) at 40 °C for 2 h at 100 rpm to dissolve the substrates. After this time, the samples were cooled and left in the refrigerator for 20 h. The mixtures were centrifuged using a Centurion Scientific K3 series centrifuge model K2015R (Stoughton, UK) at a speed of 4800× *g* for 20 min at 4 °C to precipitate the obtained inclusion complexes. The supernatants were decanted through filter paper into clean Falcon tubes. After centrifugation, the precipitated complexes were frozen and lyophilized. The dried complexes were extracted once with 160 mL of anhydrous ethanol. For this purpose, the mixtures were shaken at room temperature for 5 min in a Lab Companion OS-4000 orbital shaker (JEIO TECH, Yuseong-gu, Daejeon, Republic of Korea) and centrifuged (4800× *g*, 10 min, 4 °C). The supernatants were decanted and combined with the first portion of aqueous supernatants. The residue after centrifugation, which consisted of the obtained inclusion complexes, was dried in a stream of nitrogen using an N118LA generator (Peak Scientific Instruments Ltd., Inchinnan, Scotland, UK).

#### 3.2.3. Synthesis of ACTICOA Inclusion Complexes with β-CD or HP-β-CD Using Reactive Extraction

ACTICOA inclusion complexes with cyclodextrins were obtained by reactive extraction according to the modified method of El Darra et al. [19]. For this purpose, 0.5 g of ACTICOA was weighed, to which 20 mL of a 1.0% β-CD or HP-β-CD solution was added and shaken in an SV 1422 Memmert water bath (Schwabach, Germany) for 2 h at 40 °C. After this time, the samples were filtered through filter paper and stored at −20 °C, and then freeze-dried (−50 °C, 0.9 mbar) using a DELTA 1-24 LSC Christ freeze dryer (Martin Christ, Osterode am Harz, Germany).

#### 3.2.4. Preparation of Alginate–Chitosan Capsules Using Method 1 or Method 2

In order to obtain empty A-Ch capsules and A-Ch capsules with a bioactive substance (EACTICOA; (+)-C, Q, or GA), methods described by Pasukamonset et al. [69] were used.

In method 1, 5 mL of a 1% sodium alginate solution was prepared and mixed with 5 mL of a bioactive substance with a concentration of 3 mg/mL. Then, the resulting mixture was added dropwise to a flask containing 10 mL of 1% calcium chloride solution mixed with 5 mL of 0.5% chitosan solution dissolved in 1% acetic acid solution. As a result of combining the substances, spherical-shaped microspheres were formed, which were then mixed in a Lab Companion OS-4000 orbital shaker (JEIO TECH, Yuseong-gu, Daejeon, Republic of Korea) at room temperature at the speed of 100 rpm for 20 min. The obtained microspheres were filtered through a paper filter, and the residue on the filter was washed with deionized water. The microcapsules were left to dry for 15 min.

In method 2, a mixture of 5 mL of 1% sodium alginate solution mixed with 5 mL of bioactive substance at a concentration of 3 mg/mL was combined with 5 mL of 1% calcium chloride solution. Then, this was stirred in an orbital shaker at room temperature at the speed of 100 rpm for 2 h. The obtained microspheres were filtered through filter paper and the residue was washed with deionized water. After 15 min, the capsules were transferred to 5 mL of 0.5% chitosan solution dissolved in 1% acetic acid solution. The mixture was shaken (room temperature, 30 min, 100 rpm) on an orbital shaker and then left to dry for 15 min.

### 3.3. Preparation of Drinking Yogurt

The yogurt was prepared under laboratory conditions using a BROWIN Llp yogurt maker (Łódź, Poland). Milk with a fat content of 3.2% (brand “MU”, District Dairy Cooperative WART-MILK, Sieradz, Poland) was heated to 44 °C and placed in a yogurt preparation container. Freeze-dried bacteria culture (0.020 g/L) (*Streptococcus salivarius* subsp. *thermophilus*, *Lactobacillus delbrueckii* subsp. *bulgaricus*) YO-122 (Serowar C.P., Szczecin, Poland) was dissolved in milk heated to 44 °C. The bacterial starter was poured into containers of a yogurt pot and incubated for 4.5 h for fermentation until a clot of milk was obtained. The obtained yogurt was cooled to 4 °C and stored at a refrigerated temperature of 4 °C until further analysis.

Subsequently, the yogurt was divided into smaller samples, and the following biological activity preparations described below were added to them and stored under the refrigeration conditions mentioned above for 14 days. 

### 3.4. Adding of Bioactive Preparations to Yogurt 

The obtained yogurts were enriched with a 1% (*w*/*v*) addition of cocoa phenolic compounds, including ACTICOA cocoa powder (Barry Callebaut Polska Ltd., Łódź, Poland) and the extract obtained from it, selected polyphenolic compounds, i.e., (+)-catechin (Sigma-Aldrich, Poznań, Poland), quercetin (Sigma-Aldrich, Poland) and gallic acid (Sigma-Aldrich, Poland), obtained cocoa phenolic compounds inclusion complexes with β-cyclodextrin (β-CD) and its derivative hydroxypropyl-β-cyclodextrin (HP-β-CD) obtained by the classic method and as a result of reactive extraction and alginate–chitosan capsules with EACTICOA and selected cocoa phenolic compounds obtained using two methods (v.1, v.2).

### 3.5. Methods

#### 3.5.1. Microbiological Analysis of Yogurt

Microbiological analysis included determination of the number of lactic acid bacteria (*Streptococcus salivarius* subsp. *thermophilus*, *Lactobacillus delbrueckii* subsp. *bulgaricus*) after fermentation and after storage. The number of bacteria was determined by the plate method. A total of 1 g of yogurt was suspended in 99 mL of physiological saline (0.9% NaCl, *w*/*v*). Ten-fold dilutions were then made and plated onto Petri dishes. For the determination of *Lactobacillus delbrueckii* subsp. *bulgaricus*, MRS Agar (Merck, Darmstadt, Germany) was used, while for the determination of *Streptococcus salivarius* subsp. *thermophilus*, Agar M-17 medium (BTL. Ltd., Łódź, Poland) was used. *Lactobacillus delbrueckii* subsp. *bulgaricus* was incubated at 37 °C for 96 h. In the case of *Streptococcus salivarius* subsp. *thermophilus* incubation was carried out at 44–45 °C for 96 h.

Yogurts after fermentation and after 14 days of storage with and without polyphenol preparations were analyzed. The results of the microbial analysis are presented as log (cfu/mL) ± SD (standard deviation) (logarithmic units—LU). The test was performed in three independent replications.

#### 3.5.2. UHPLC-DAD-ESI–MS/MS Analysis of Phenolic Compounds

The qualitative and quantitative composition of phenolic compounds and their identification were performed in accordance with the methodology described by Żyżelewicz et al. [33,68].

#### 3.5.3. Efficiency of Encapsulate Obtaining

The efficiency of encapsulate obtaining was calculated according to the procedure of Żyżelewicz et al. [33] based on the concentration of bioactive compound (e.g., (+)-C) in the reaction solution before obtaining capsules (g/mL) and the concentration of this component (e.g., (+)-C) in free form (g/mL) after the encapsulation process, determined by the UHPLC-DAD-ESI-MS/MS method [33].

#### 3.5.4. Total Phenolic Content Determination

The determination of the total polyphenol content (TPC) was performed according to the procedure described by Belščak et al. [70]. A calibration curve determined for (+)-catechin as a standard was used to calculate TPC in the tested samples. The results were expressed as milligrams of (+)-catechin per gram of the lyophilized preparation (mg CE/g of the product).

#### 3.5.5. Antioxidant Activity Determination

In vitro methods were used to determine the antioxidant properties of the tested samples. The DPPH free radical scavenging capacity was determined as described by Schinella et al. [71]. The absorbance of the prepared solutions was measured at the wavelength of 517 nm using a Shimadzu UV-1800 spectrophotometer (Tokyo, Japan). The results were presented in the form of the EC50 parameter and expressed in mg of sample per mg DPPH radical (mg/mg DPPH). In turn, the ability to scavenge the free cation radical ABTS was determined using the method of Belščak et al. [70]. The absorbance of the prepared solutions was measured at the wavelength of 734 nm. The results were presented in the form of the EC50 parameter and expressed in mg of sample per mg ABTS radical (mg/mg ABTS). The determination of the iron(III) ion reduction capacity (FRAP test) was performed using the procedure described by Vignoli et al. [72]. The absorbance of properly prepared test samples was measured at the wavelength of 593 nm. The assay results were given in µM Trolox/g.

#### 3.5.6. Statistical Analysis

All experiments on phenolic compound preparations and yogurts were carried out in triplicate on two separate occasions (*n* = 6), and all values were expressed as mean values with standard deviation (SD). The microbiological data represented triplicate observations in each independent experiment (*n* = 3). Statistica 13.0 software (StatSoft, Inc., Tulsa, OK, USA) was used for the statistical data analyses. The significant differences were estimated by one-way analysis of variance (ANOVA) followed by Tukey’s Honest Significant Difference (HSD) test. Differences were considered to be significant when *p*-values were less than 0.05 (*p* < 0.05).

Statistical analysis of the results obtained in microbiological tests was performed using one-way ANOVA with Bonferroni post-hoc test (*p* ≤ 0.017).

## 4. Conclusions

The results obtained in this study clearly showed that yogurts enriched with cocoa phenolic compound preparations were characterized by different antioxidant activities. The biological properties of the obtained products depended on the type of active substance included in the formulation, the type of coating material and the method of obtaining complexes and capsules. The results showed that the enrichment of yogurts with selected encapsulates rich in phenolic compounds found in ACTICOA can have a beneficial effect on the number of lactic acid bacteria in yogurts. It can be stated that the encapsulation process of phenolic compounds protects the lactic acid bacteria present in the yogurt from the adverse antagonistic effects of these compounds. The highest content of phenolic compounds was observed in yogurts enriched with free phenolic compounds, followed by EACTICOA and its inclusion complexes with β-CD or HP-β-CD. Yogurts fortified with free phenolic compounds, EACTICOA and inclusion complexes of these substances with CDs showed the strongest antioxidant activity measured using three different in vitro assays. In general, the enrichment of yogurts with inclusion complexes of (+)-C, Q, GA and EACTICOA with cyclodextrins proved to be the most favorable approach to increase the phenolic compounds content and antioxidant activity of fresh and stored drinking yogurts enriched with the tested encapsulates.

In further studies, it seems reasonable to investigate the possibility of effective enrichment of other food products with the investigated preparations. Moreover, given the changes that the phenolic compounds present in the tested preparations may undergo after ingestion, further research is needed to determine the modifications that occur in the content of the phenolic compounds and in their biological activity during the simulated digestion and as a result of interactions with the gut microflora.

## Figures and Tables

**Table 1:** Composition and concentration of phenolic compounds in ACTICOA and EACTICOA.

Tested Parameter	ACTICOA	EACTICOA
(+)–Catechin	3.47 ± 0.03 ^a^	17.33 ± 0.12 ^b^
(−)–Epicatechin	18.59 ± 0.12 ^a^	107.42 ± 0.13 ^b^
Procyanidin B2	7.56 ± 0.10 ^a^	46.94 ± 0.05 ^b^
Procyanidin C1	5.88 ± 0.05 ^a^	29.72 ± 0.08 ^b^
Total flavan-*3*-ols	35.49 ± 0.21 ^a^	201.39 ± 0.15 ^b^
Gallic acid	0.11 ± 0.01 ^a^	0.63 ± 0.03 ^b^
Protocatechuic acid	0.11 ± 0.02 ^a^	0.74 ± 0.02 ^b^
Caffeic acid aspartate	1.47 ± 0.13 ^a^	6.72 ± 0.05 ^b^
*p*-Coumaric acid aspartate	0.04 ± 0.01 ^a^	0.18 ± 0.02 ^b^
Total phenolic acids	1.72 ± 0.06 ^a^	8.26 ± 0.10 ^b^
Total phenolic compounds	37.21 ± 0.74 ^a^	209.65 ± 0.70 ^b^
TPC (mg CE/g)	228.85 ± 0.15 ^a^	435.14 ± 0.18 ^b^

Data presented as mean ± SD (*n* = 6); ACTICOA—ACTICOA cocoa powder; EACTICOA—extract of ACTICOA cocoa powder; (+)-C—(+)–catechin; TPC—total phenolic compounds content determined by the Folin–Ciocalteu spectrophotometric method; Values in rows with the same superscript (a,b) are not significantly different (Tukey’s HSD test, *p* < 0.05).

**Table 2:** Formation efficiency, composition and concentration of phenolic compounds and total content of phenolic compounds of tested inclusion complexes.

Tested Parameter	Type of Encapsulate							
	(+)-C-β-CD	Q-β-CD	GA-β-CD	(+)-C-HP-β-CD	Q-HP-β-CD	GA-HP-β-CD	ACTICOA-β-CD(RE)	ACTICOA-HP-β-CD(RE)
Obtaining efficiency (%)	67.81 ± 0.79 ^f^	55.75 ± 0.67 ^c^	67.54 ± 0.54 ^e^	98.90 ± 1.10 ^h^	62.46 ± 1.54 ^d^	98.20 ± 1.34 ^g^	42.97 ± 4.01 ^a^	50.60 ± 1.50 ^b^
Concentration of phenolic compounds in the encapsulate (mg/g)								
Flavonoids								
(+)–Catechin	155.68 ± 0.23 ^c^	nd	nd	184.87 ± 0.31 ^d^	nd	nd	1.15 ± 0.01 ^b^	0.63 ± 0.02 ^a^
(−)–Epicatechin	nd	nd	nd	nd	nd	nd	8.71 ± 0.04 ^b^	5.45 ± 0.06 ^a^
Procyanidin B2	nd	nd	nd	nd	nd	nd	3.14 ± 0.03 ^b^	1.82 ± 0.01 ^a^
Procyanidin C1	nd	nd	nd	nd	nd	nd	4.62 ± 0.01 ^b^	2.74 ± 0.06 ^a^
Quercetin	nd	135.98 ± 0.21 ^a^	nd	nd	178.98 ± 0.28 ^b^	nd	nd	nd
Total flavonoids	155.68 ± 0.23 ^d^	135.98 ± 0.21 ^c^	nd	184.87 ± 0.31 ^f^	178.98 ± 0.28 ^e^	nd	17.62 ± 0.08 ^b^	10.64 ± 0.12 ^a^
Phenolic acids								
Gallic acid	nd	nd	245.09 ± 0.25 ^a^	nd	nd	252.09 ± 0.31 ^b^	nd	nd
Protocatechuic acid	nd	nd	nd	nd	nd	nd	nd	nd
Caffeic acid aspartate	nd	nd	nd	nd	nd	nd	0.07 ± 0.02 ^b^	0.04 ± 0.02 ^a^
*p*-Coumaric acid aspartate	nd	nd	nd	nd	nd	nd	0.02 ± 0.01 ^a^	0.01 ± 0.01 ^a^
Total phenolic acids	nd	nd	245.09 ± 0.25 ^c^	nd	nd	252.09 ± 0.31 ^d^	0.09 ± 0.03 ^b^	0.06 ± 0.0 ^a^
Total phenolic compounds	155.68 ± 0.23 ^d^	135.98 ± 0.21 ^c^	245.09 ± 0.25 ^g^	184.87 ± 0.31 ^f^	178.98 ± 0.28 ^e^	252.09 ± 0.31 ^b^	17.79 ± 0.15 ^b^	10.70 ± 0.12 ^a^
TPC (mg CE/g)	202.26 ± 0.18 ^e^	211.53 ± 0.13 ^f^	287.09 ± 0.16 ^g^	165.03 ± 0.19 ^c^	294.89 ± 0.23 ^h^	197.94 ± 0.17 ^d^	109.81 ± 0.19 ^b^	101.06 ± 0.16 ^a^

Data presented as mean ± SD (*n* = 6); Values in rows with the same superscript (a–h) are not significantly different (Tukey’s HSD test, *p* < 0.05). nd—not detected; ACTICOA—ACTICOA cocoa powder; EACTICOA—extract of ACTICOA cocoa powder; (+)-C—(+)–catechin; Q—quercetin; GA—gallic acid; β-CD—β-cyclodextrin; HP-β-CD—hydroxypropyl-β-cyclodextrin; (+)-C-β-CD—inclusion complex of (+)-catechin with β-CD; Q-β-CD—inclusion complex of quercetin with β-CD; GA-β-CD—inclusion complex of gallic acid with β-CD; (+)-C-HP-β-CD—inclusion complex of (+)-catechin with HP-β-CD; Q-HP-β-CD—inclusion complex of quercetin with HP-β-CD; GA-HP-β-CD—inclusion complex of gallic acid with HP-β-CD; ACTICOA-β-CD(RE)—inclusion complex of ACTICOA with β-CD obtained as a result of reactive extraction; ACTICOA-HP-β-CD(RE)—inclusion complex of ACTICOA with HP-β-CD obtained as a result of reactive extraction; TPC—total phenolic compounds content determined by the Folin–Ciocalteu spectrophotometric method.

**Table 3:** Formation efficiency, composition and concentration of phenolic compounds and total content of phenolic compounds of tested alginate–chitosan capsules.

Tested Parameter	Type of Encapsulate							
	A-Ch-EACTICOA v.1	A-Ch-EACTICOA v.2	A-Ch-(+)-C v.1	A-Ch-(+)-C v.2	A-Ch-Q v.1	A-Ch-Q v.2	A-Ch-GA v.1	A-Ch-GA v.2
Obtaining efficiency (%)	95.67 ± 0.38 ^b^	94.78 ± 0.29 ^a^	98.34 ± 0.23 ^d^	99.45 ± 0.25 ^g^	99.87 ± 0.23 ^h^	97.67 ± 0.78 ^c^	98.64 ± 0.56 ^e^	98.89 ± 0.29 ^f^
Concentration of phenolic compounds in the encapsulate (mg/g)								
Flavonoids								
(+)–Catechin	1.12 ± 0.03 ^b^	1.06 ± 0.01 ^a^	53.42 ± 0.10 ^c^	54.10 ± 0.12 ^d^	nd	nd	nd	nd
(−)–Epicatechin	5.91 ± 0.03 ^b^	5.80 ± 0.03 ^a^	nd	nd	nd	nd	nd	nd
Procyanidin B2	3.22 ± 0.02 ^a^	3.21 ± 0.01 ^a^	nd	nd	nd	nd	nd	nd
Procyanidin C1	1.61 ± 0.01 ^a^	1.62 ± 0.02 ^a^	nd	nd	nd	nd	nd	nd
Quercetin	nd	nd	nd	nd	62.98 ± 0.16 ^b^	57.54 ± 0.27 ^a^	nd	nd
Total flavonoids	11.86 ± 0.09 ^a^	11.69 ± 0.07 ^b^	53.42 ± 0.10 ^c^	54.10 ± 0.12 ^d^	62.98 ± 0.16 ^f^	57.54 ± 0.27 ^e^	nd	nd
Phenolic acids								
Gallic acid	nd	nd	nd	nd	nd	nd	55.09 ± 0.10 ^b^	45.37 ± 0.13 ^a^
Caffeic acid aspartate	0.06 ± 0.01 ^a^	0.06 ± 0.01 ^a^	nd	nd	nd	nd	nd	nd
*p*-Coumaric acid aspartate	0.02 ± 0.01 ^a^	0.02 ± 0.01 ^a^	nd	nd	nd	nd	nd	nd
Total phenolic acids	0.08 ± 0.02 ^a^	0.08 ± 0.02 ^a^	nd	nd	nd	nd	nd	nd
Total phenolic compounds	11.94 ± 0.11 ^b^	11.77 ± 0.10 ^a^	53.42 ± 0.10 ^d^	54.10 ± 0.12 ^e^	62.98 ± 0.16 ^h^	57.54 ± 0.27 ^g^	55.09 ± 0.10 ^f^	45.37 ± 0.13 ^c^
TPC (mg CE/g)	32.46 ± 0.09 ^c^	29.23 ± 0.08 ^b^	73.50 ± 0.16 ^f^	25.69 ± 0.05 ^a^	189.63 ± 0.19 ^h^	159.71 ± 0.08 ^g^	69.40 ± 0.11 ^e^	35.73 ± 0.04 ^d^

Data presented as mean ± SD (*n* = 6); Values in rows with the same superscript (a–h) are not significantly different (Tukey’s HSD test, *p* < 0.05). nd—not detected; A-Ch-EACTICOA v.1—alginate–chitosan capsules with EACTICOA obtained according to method 1; A-Ch-EACTICOA v.2—alginate–chitosan capsules with EACTI-COA obtained according to method 2; A-Ch-(+)-C v.1—alginate–chitosan capsules with (+)-catechin obtained according to method 1; A-Ch-(+)-C v.2—alginate–chitosan capsules with (+)-catechin obtained according to method 2; A-Ch-Q v.1—alginate–chitosan capsules with quercetin obtained according to method 1; A-Ch-Q v.2—alginate–chitosan capsules with quercetin obtained according to method 2; A-Ch-GA v.1—alginate–chitosan capsules with gallic acid obtained according to method 1; A-Ch-GA v.2—alginate–chitosan capsules with gallic acid obtained according to method 2; TPC—total phenolic compounds content determined by the Folin-Ciocalteu spectrophotometric method.

**Table 4:** Antioxidant activity of tested preparations.

Preparation	DPPH EC50 (mg/mg DPPH)	ABTS EC50 (mg/mg ABTS)	FRAP (µM Trolox/g)
ACTICOA	0.55 ± 0.02 ^c^	0.32 ± 0.03 ^f^	1105.32 ± 1.06 ^p^
EACTICOA	0.20 ± 0.01 ^b^	0.15 ± 0.02 ^c^	2804.29 ± 1.12 ^r^
(+)-C	0.23 ± 0.01 ^b^	0.05 ± 0.01 ^a^	4801.51 ± 1.09 ^s^
Q	0.21 ± 0.01 ^b^	0.12 ± 0.02 ^b^	6616.17 ± 1.16 ^t^
GA	0.15 ± 0.01 ^a^	0.15 ± 0.01 ^c^	7204.89 ± 1.08 ^u^
(+)-C-β-CD	2.09 ± 0.02 ^g^	0.48 ± 0.02 ^h^	477.49 ± 2.21 ^g^
Q-β-CD	2.07 ± 0.01 ^g^	0.35 ± 0.01 ^g^	596.78 ± 1.45 ^i^
GA-β-CD	1.56 ± 0.01 ^d^	0.21 ± 0.02 ^e^	778.56 ± 1.26 ^m^
(+)-C-HP-β-CD	4.01 ± 0.03 ^j^	0.22 ± 0.01 ^e^	580.83 ± 1.98 ^h^
Q-HP-β-CD	2.56 ± 0.02 ^i^	0.20 ± 0.02 ^d^	657.98 ± 1.18 ^j^
GA-HP-β-CD	1.89 ± 0.01 ^f^	0.18 ± 0.01 ^d^	845.74 ± 1.07 ^o^
ACTICOA-β-CD(RE)	1.65 ± 0.01 ^e^	1.08 ± 0.01 ^i^	819.72 ± 1.21 ^n^
ACTICOA-HP-β-CD(RE)	2.21 ± 0.02 ^h^	1.45 ± 0.02 ^j^	1128.2 ± 1.08 ^p^
A-Ch-EACTICOA v.1	12.08 ± 0.09	9.89 ± 0.03 ^s^	200.52 ± 1.06 ^c^
A-Ch-EACTICOA v.2	9.92 ± 0.07 ^o^	6.67 ± 0.03 ^p^	180.75 ± 1.09 ^a^
A-Ch-(+)-C v.1	9.98 ± 0.05 ^n^	6.75 ± 0.01 ^r^	729.42 ± 1.11 ^l^
A-Ch-(+)-C v.2	9.09 ± 0.08 ^m^	5.60 ± 0.02 ^o^	459.73 ± 1.05 ^f^
A-Ch-Q v.1	8.66 ± 0.06 ^l^	2.25 ± 0.01 ^l^	337.95 ± 1.01 ^e^
A-Ch-Q v.2	7.68 ± 0.04 ^k^	1.87 ± 0.02 ^k^	675.89 ± 1.12 ^k^
A-Ch-GA v.1	10.98 ± 0.07 ^p^	3.38 ± 0.02 ^n^	206.54 ± 1.07 ^d^
A-Ch-GA v.2	13.64 ± 0.05 ^r^	2.80 ± 0.01 ^m^	186.17 ± 1.04 ^b^

Data presented as mean ± SD (*n* = 6); Values in columns with the same superscript (a–u) are not significantly different (Tukey’s HSD test, *p* < 0.05). ACTICOA—ACTICOA cocoa powder; EACTICOA—extract of ACTICOA cocoa powder; (+)-C—(+)–catechin; Q—quercetin; GA—gallic acid; β-CD—β-cyclodextrin; HP-β-CD—hydroxypropyl-β-cyclodextrin; (+)-C-β-CD—inclusion complex of (+)-catechin with β-CD; Q-β-CD—inclusion complex of quercetin with β-CD; GA-β-CD—inclusion complex of gallic acid with β-CD; (+)-C-HP-β-CD—inclusion complex of (+)-catechin with HP-β-CD; Q-HP-β-CD—inclusion complex of quercetin with HP-β-CD; GA-HP-β-CD—inclusion complex of gallic acid with HP-β-CD; ACTICOA-β-CD(RE)—inclusion complex of ACTICOA with β-CD obtained as a result of reactive extraction; ACTICOA-HP-β-CD(RE)—inclusion complex of ACTICOA with HP-β-CD obtained as a result of reactive extraction; A-Ch-EACTICOA v.1—alginate–chitosan capsules with EACTICOA obtained according to method 1; A-Ch-EACTICOA v.2—alginate–chitosan capsules with EACTICOA obtained according to method 2; A-Ch-(+)-C v.1—alginate–chitosan capsules with (+)-catechin obtained according to method 1; A-Ch-(+)-C v.2—alginate–chitosan capsules with (+)-catechin obtained according to method 2; A-Ch-Q v.1—alginate–chitosan capsules with quercetin obtained according to method 1; A-Ch-Q v.2—alginate–chitosan capsules with quercetin obtained according to method 2; A-Ch-GA v.1—alginate–chitosan capsules with gallic acid obtained according to method 1; A-Ch-GA v.2—alginate–chitosan capsules with gallic acid obtained according to method 2.

**Table 5:** The content of lactic acid bacteria (*Lactobacillus* spp., *Streptococcus* spp.) in tested yogurts.

Kind of Yogurt/Bioactive Additive	*Lactobacillus* spp. Log (cfu/mL) ± SD	*Streptococcus* spp. Log (cfu/mL) ± SD
CS after fermentation	7.7 ± 0.43 ^a^	9.3± 0.07 ^e^
CS after 14 days storage	6.5 ± 0.76	8.8± 0.23 *
Yogurts with additions after 14 days of storage		
ACTICOA	5.4 ± 0.33 ^b^	7.6 ± 0.00 ^d#^
EACTICOA	5.9 ± 0.68 ^b^	8.0 ± 0.11 ^d^
(+)-C	5.7 ± 0.43 ^b^	5.8 ± 0.00 ^d#^
Q	7.0 ± 0.66	7.4 ± 0.40 ^d^
GA	3.5 ± 1.33 ^c^	5.2 ± 0.00 ^d#^
(+)-C-β-CD	5.5 ± 0.18 ^b^	7.5 ± 0.99
Q-β-CD	6.7 ± 0.57	8.1 ± 0.11 ^d^
GA-β-CD	6.9 ± 0.27	8.1 ± 0.04 ^d^
(+)-C-HP-β-CD	5.4 ± 0.18 ^b^	6.5 ± 0.50 ^d#^
Q-HP-β-CD	5.6 ± 0.00 ^b^	5.7 ± 0.24 ^d#^
GA-HP-β-CD	5.3 ± 0.00 ^b^	7.8 ± 0.13 ^d^
ACTICOA-β-CD(RE)	5.6 ± 0.60	7.5 ± 0.00 ^d#^
ACTICOA-HP-β-CD(RE)	6.3 ± 1.0	7.7 ± 0.93
A-Ch-EACTICOA v.1	5.7 ± 0.00 ^b^	8.2 ± 0.04 ^d^
A-Ch-EACTICOA v.2	5.5 ± 0.28 ^b^	8.2 ± 0.15 ^d^
A-Ch-(+)-C v.1	6.0 ± 0.54 ^b^	8.2 ± 0.43
A-Ch-(+)-C v.2	7.2 ± 0.23	7.6 ± 1.34
A-Ch-Q v.1	7.5 ± 0.04	8.7 ± 0.39
A-Ch-Q v.2	5.7 ± 0.48	8.1 ± 0.36
A-Ch-GA v.1	7.0 ± 0.73	8.5 ± 0.25
A-Ch-GA v.2	6.7 ± 0.11	8.3 ± 0.12 ^d^

SD—standard deviation; a–c—statistical differences between the yogurt after fermentation and each enriched yogurt and yogurt without additives after 14 days of fermentation (for *Lactobacillus* spp.); d,e—statistical differences between the yogurt after fermentation and each fortified yogurt and yogurt without additives after 14 days of fermentation (for *Streptococcus* spp.); *, ^#^—statistical differences between pure yogurt after 14 days storage and each fortified yogurt and yogurt after fermentation (for *Streptococcus* spp.); Values are presented as mean ± SD (*n* = 6); Statistical analysis was performed using One-way ANOVA, Bonferroni post-hock test (*p* ≤ 0.017); CS—control sample (yogurt without additives). Abbreviations of tested samples as in Table 4.

## Data Availability

The original contributions presented in the study are included in the article, further inquiries can be directed to the corresponding author.

## Supplementary Materials

The following supporting information can be downloaded at: https://www.mdpi.com/article/10.3390/molecules29143344/s1. Figure S1: UHPLC-DAD chromatogram of the phenolic compounds recorded at 280 nm in ACTICOA cocoa powder sample; Figure S2: UHPLC-DAD chromatogram of the phenolic compounds recorded at 280 nm in EACTICOA sample; Figure S3: UHPLC-DAD chromatogram of (+)–catechin recorded at 280 nm; Figure S4: UHPLC-DAD chromatogram of quercetin recorded at 280 nm; Figure S5: UHPLC-DAD chromatogram of gallic acid recorded at 280 nm; Figure S6: UHPLC-DAD chromatogram of the phenolic compounds recorded at 280 nm in inclusion complex of (+)-catechin with β-CD; Figure S7: UHPLC-DAD chromatogram of the phenolic compounds recorded at 280 nm in inclusion complex of quercetin with β-CD; Figure S8: UHPLC-DAD chromatogram of the phenolic compounds recorded at 280 nm in inclusion complex of gallic acid with β-CD; Figure S9: UHPLC-DAD chromatogram of the phenolic compounds recorded at 280 nm in inclusion complex of (+)-catechin with HP-β-CD; Figure S10: UHPLC-DAD chromatogram of the phenolic compounds recorded at 280 nm in inclusion complex of quercetin with HP-β-CD; Figure S11: UHPLC-DAD chromatogram of the phenolic compounds recorded at 280 nm in inclusion complex of gallic acid with HP-β-CD; Figure S12: UHPLC-DAD chromatogram of the phenolic compounds recorded at 280 nm in inclusion complex of ACTICOA with β-CD obtained as a result of reactive extraction sample; Figure S13: UHPLC-DAD chromatogram of the phenolic compounds recorded at 280 nm in inclusion complex of ACTICOA with HP-β-CD obtained as a result of reactive extraction sample; Figure S14: UHPLC-DAD chromatogram of the phenolic compounds recorded at 280 nm in alginate-chitosan capsules with EACTICOA obtained according to method 1; Figure S15: UHPLC-DAD chromatogram of the phenolic compounds recorded at 280 nm in alginate-chitosan capsules with EACTICOA obtained according to method 2; Figure S16: UHPLC-DAD chromatogram of the phenolic compounds recorded at 280 nm in i alginate-chitosan capsules with (+)-catechin obtained according to method 1; Figure S17: UHPLC-DAD chromatogram of the phenolic compounds recorded at 280 nm in i alginate-chitosan capsules with (+)-catechin obtained according to method 2; Figure S18: UHPLC-DAD chromatogram of the phenolic compounds recorded at 280 nm in i alginate-chitosan capsules with quercetin obtained according to method 1; Figure S19: UHPLC-DAD chromatogram of the phenolic compounds recorded at 280 nm in i alginate-chitosan capsules with quercetin obtained according to method 2; Figure S20: UHPLC-DAD chromatogram of the phenolic compounds recorded at 280 nm in i alginate-chitosan capsules with gallic acid obtained according to method 1; Figure S21: UHPLC-DAD chromatogram of the phenolic compounds recorded at 280 nm in i alginate-chitosan capsules with gallic acid obtained according to method 2; Table S1: UHPLC-DAD-ESI-MS/MS identification of phenolic compounds in ACTICOA, EACTICOA, pure phenolic compounds and their encapsulates.
