# Supplementary material for: Effect of Encapsulated Phenolic Compounds of Cocoa on Growth of Lactic Acid Bacteria and Antioxidant Activity of Fortified Drinking Yogurt
Source: Molecules. 2024 Jul 16;29(14):3344. doi: 10.3390/molecules29143344 (PMC11279641; doi:10.3390/molecules29143344)
Supplement: Supplementary file 1 [file molecules-29-03344-s001.zip › molecules-3057662-supplementary.pdf]

Supplementary Materials

Effect of Encapsulated Phenolic Compounds of Cocoa on Growth of Lactic Acid Bacteria and Antioxidant Activity of Fortified Drinking Yogurt

Milena Rogalska [1], Joanna Oracz [1], Elżbieta Klewicka [2] and Dorota Żyżelewicz [1,*]

[1] Institute of Food Technology and Analysis, Faculty of Biotechnology and Food Sciences, Lodz University of Technology, 2/22 Stefanowskiego Street, 90-537 Lodz, Poland; milenak46@gmail.com (M.R.); joanna.oracz@p.lodz.pl (J.O.)

[2] Institute of Fermentation Technology and Microbiology, Faculty of Biotechnology and Food Sciences, Lodz University of Technology, 171/173 Wólczańska Street, 90-530 Lodz, Poland; elzbieta.klewicka@p.lodz.pl

* Correspondence: dorota.zyzelewicz@p.lodz.pl; Tel.: +48-42-631-3461

Citation: Rogalska, M.; Oracz, J.; Klewicka, E.; Żyżelewicz, D. Effect of Encapsulated Phenolic Compounds of Cocoa on Growth of Lactic Acid Bacteria and Antioxidant Activity of Fortified Drinking Yogurt. *Molecules* **2024**, *29*, 3344. https://doi.org/10.3390/molecules29143344

Academic Editor: Artur M. S. Silva

Received: 7 June 2024
Revised: 4 July 2024
Accepted: 15 July 2024
Published: 16 July 2024

Copyright: © 2024 by the authors. Licensee MDPI, Basel, Switzerland. This article is an open access article distributed under the terms and conditions of the Creative Commons Attribution (CC BY) license (https://creativecommons.org/licenses/by/4.0/).

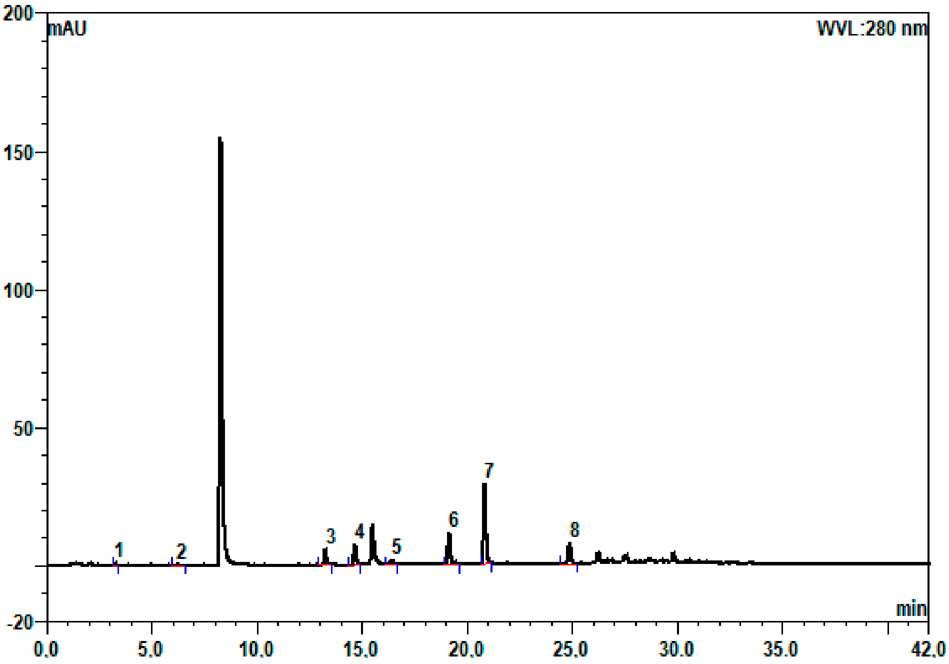

Figure S1. UHPLC-DAD chromatogram of the phenolic compounds recorded at 280 nm in ACTICOA cocoa powder sample. Peak labels: 1 - gallic acid, 2 - protocatechuic acid, 3 - caffeic acid aspartate, 4 - (+)–catechin, 5 - *p*-coumaric acid aspartate, 6 - procyanidin B2, 7 - (-)–epicatechin, 8 - procyanidin C1.

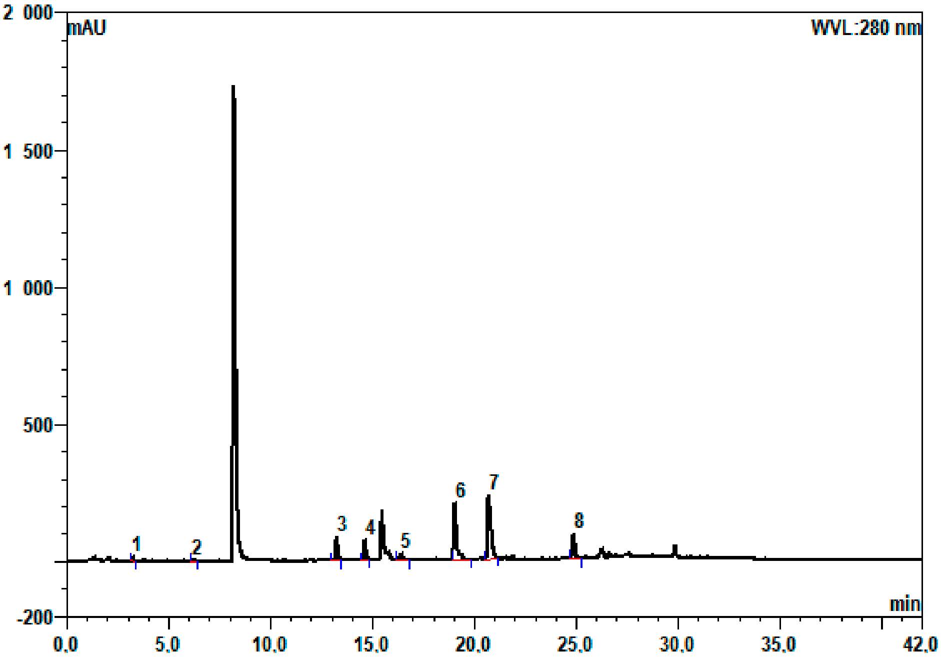

Figure S2. UHPLC-DAD chromatogram of the phenolic compounds recorded at 280 nm in EACTICOA sample. Peak labels: 1 - gallic acid, 2 - protocatechuic acid, 3 - caffeic acid aspartate, 4 - (+)–catechin, 5 - *p*-coumaric acid aspartate, 6 - procyanidin B2, 7 - (-)–epicatechin, 8 - procyanidin C1.

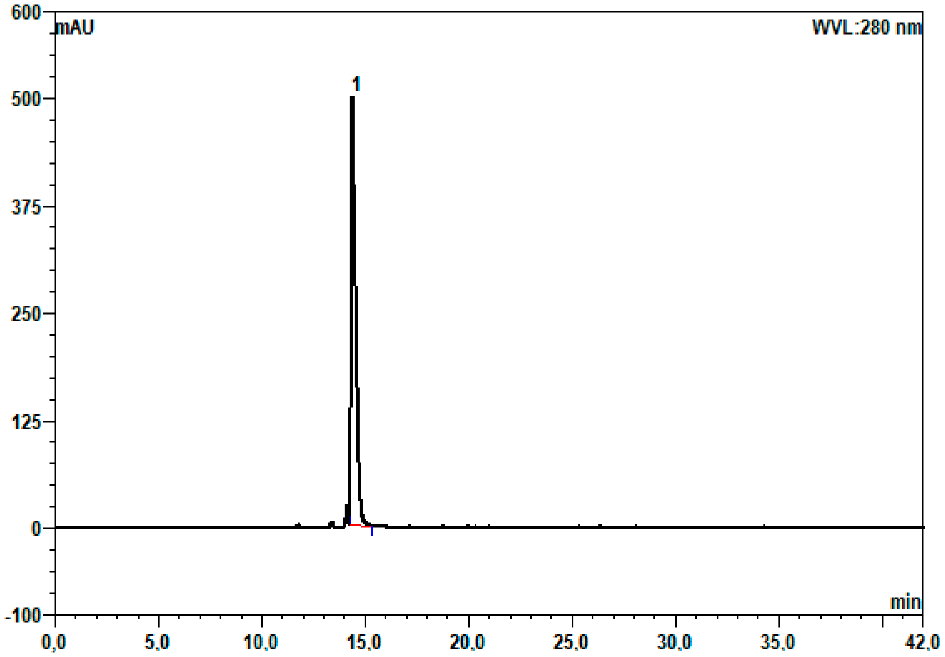

Figure S3. UHPLC-DAD chromatogram of (+)–catechin recorded at 280 nm. Peak labels: 1 - (+)–catechin.

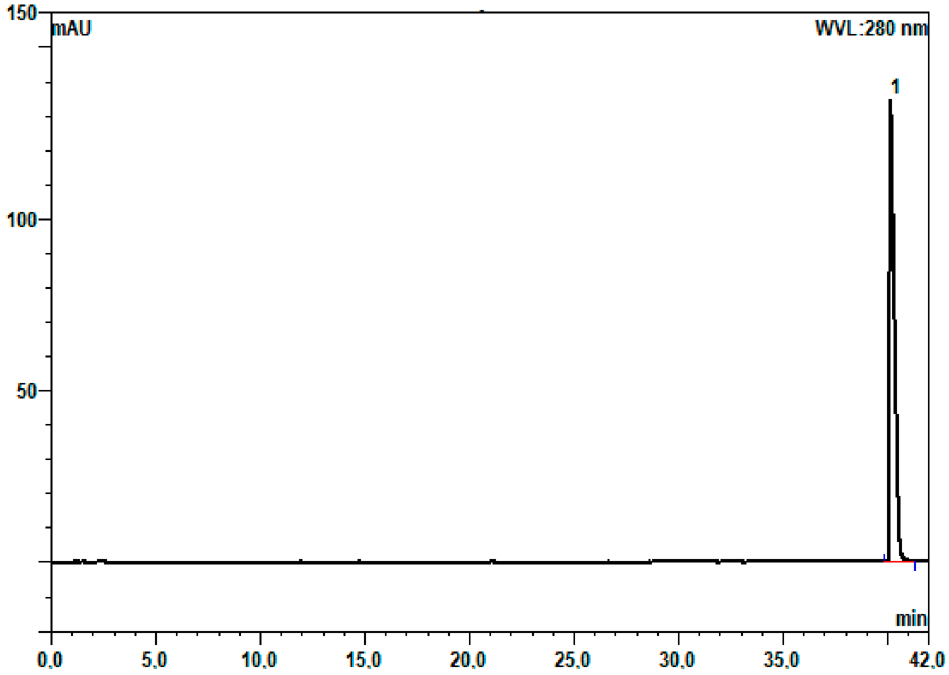

Figure S4. UHPLC-DAD chromatogram of quercetin recorded at 280 nm. Peak labels: 1 - quercetin.

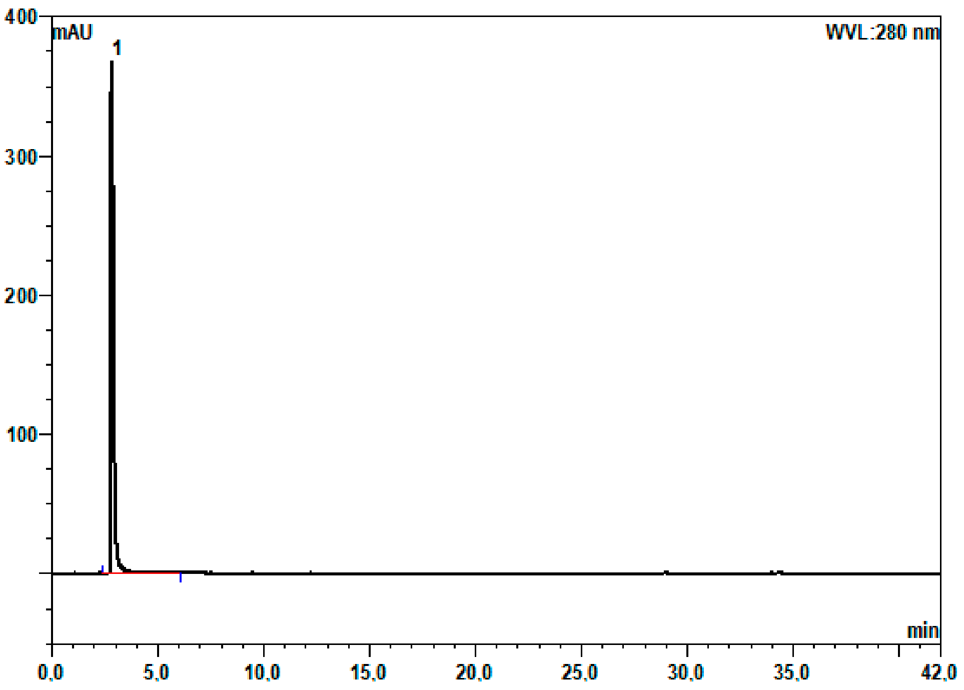

Figure S5. UHPLC-DAD chromatogram of gallic acid recorded at 280 nm. Peak labels: 1 - gallic acid.

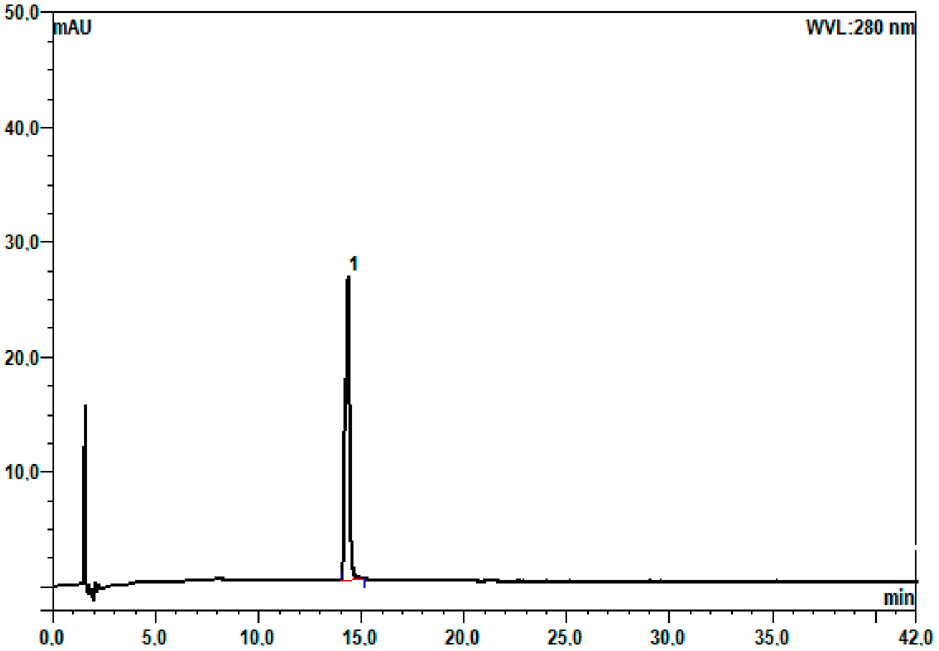

Figure S6. UHPLC-DAD chromatogram of the phenolic compounds recorded at 280 nm in inclusion complex of (+)-catechin with β-CD. Peak labels: 1 - (+)–catechin.

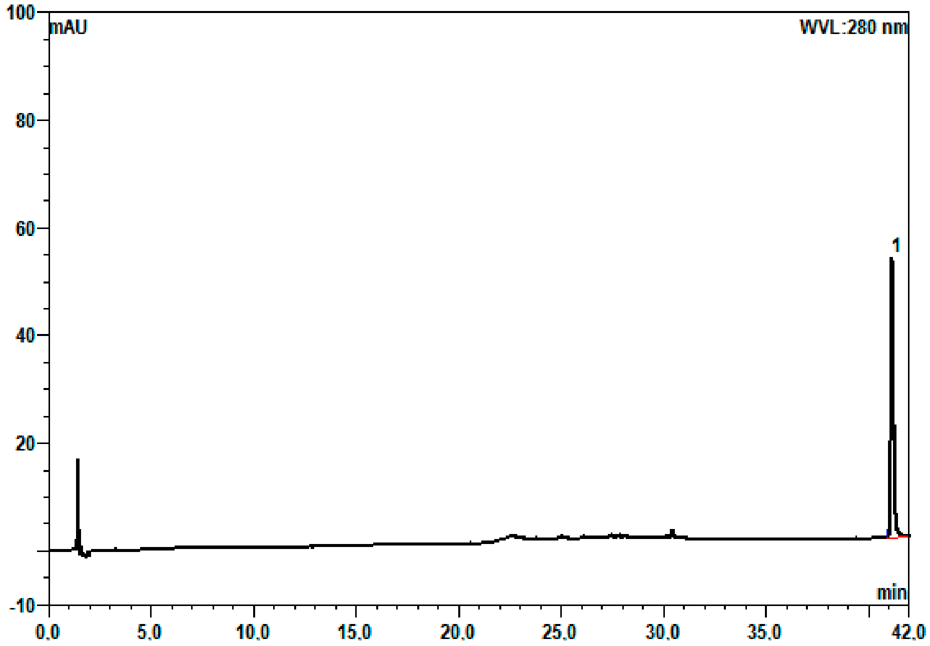

Figure S7. UHPLC-DAD chromatogram of the phenolic compounds recorded at 280 nm in inclusion complex of quercetin with β-CD. Peak labels: 1 - (+)–quercetin.

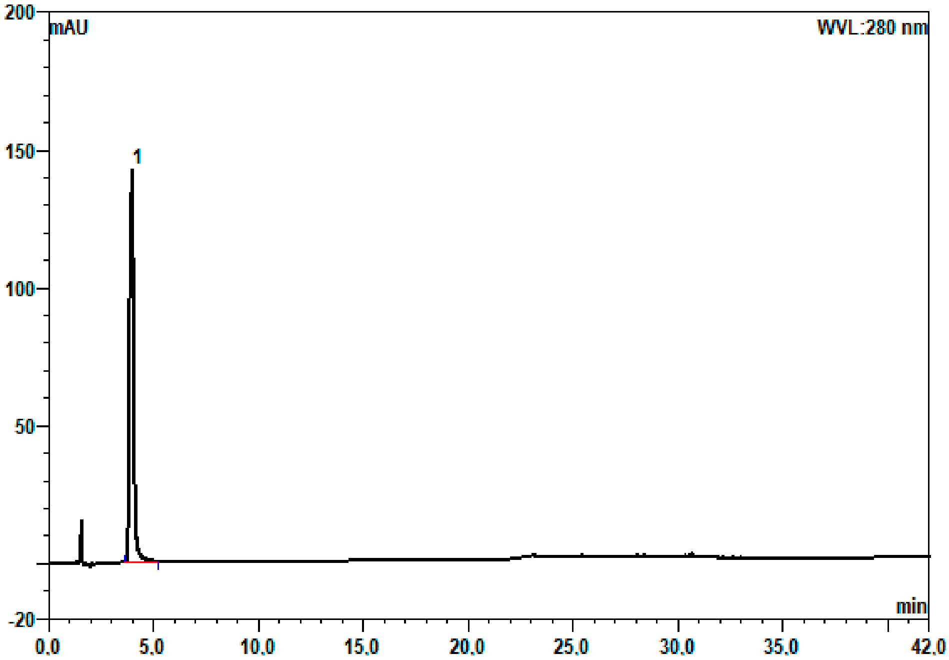

Figure S8. UHPLC-DAD chromatogram of the phenolic compounds recorded at 280 nm in inclusion complex of gallic acid with β-CD. Peak labels: 1 - gallic acid.

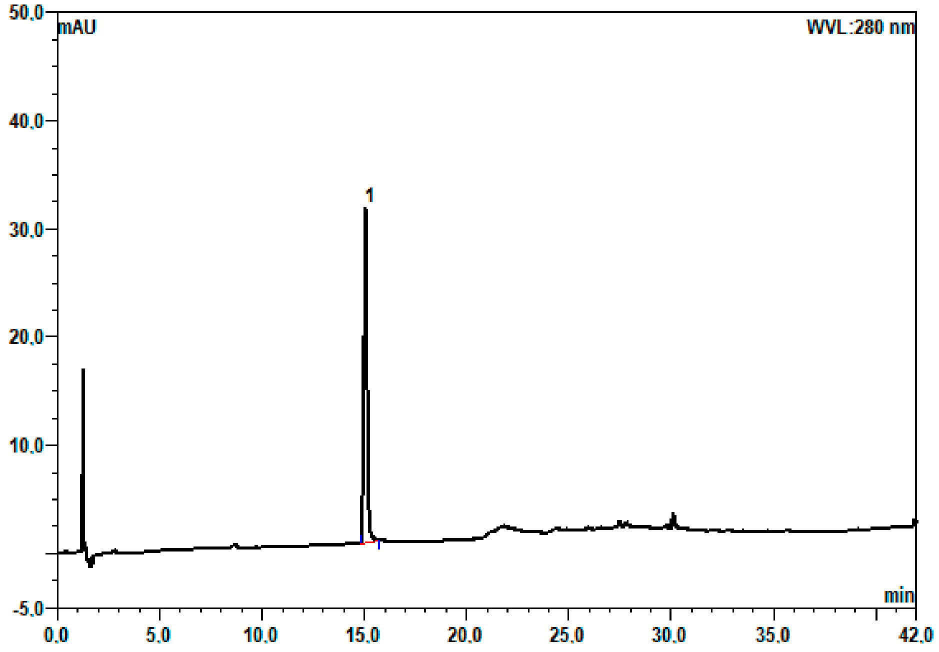

Figure S9. UHPLC-DAD chromatogram of the phenolic compounds recorded at 280 nm in inclusion complex of (+)-catechin with HP-β-CD. Peak labels: 1 - (+)–catechin.

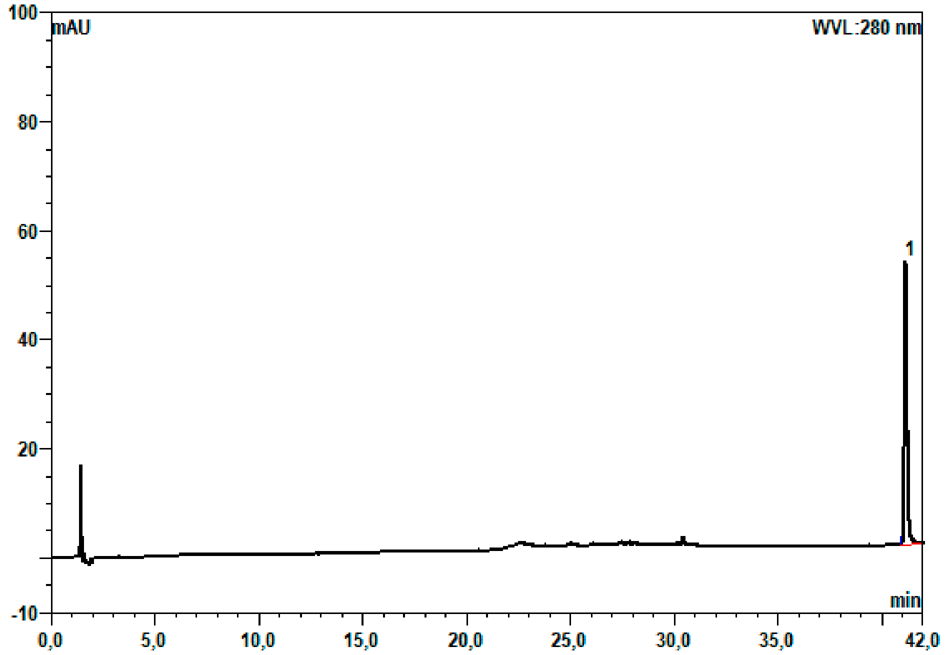

Figure S10. UHPLC-DAD chromatogram of the phenolic compounds recorded at 280 nm in inclusion complex of quercetin with HP-β-CD. Peak labels: 1 - (+)–quercetin.

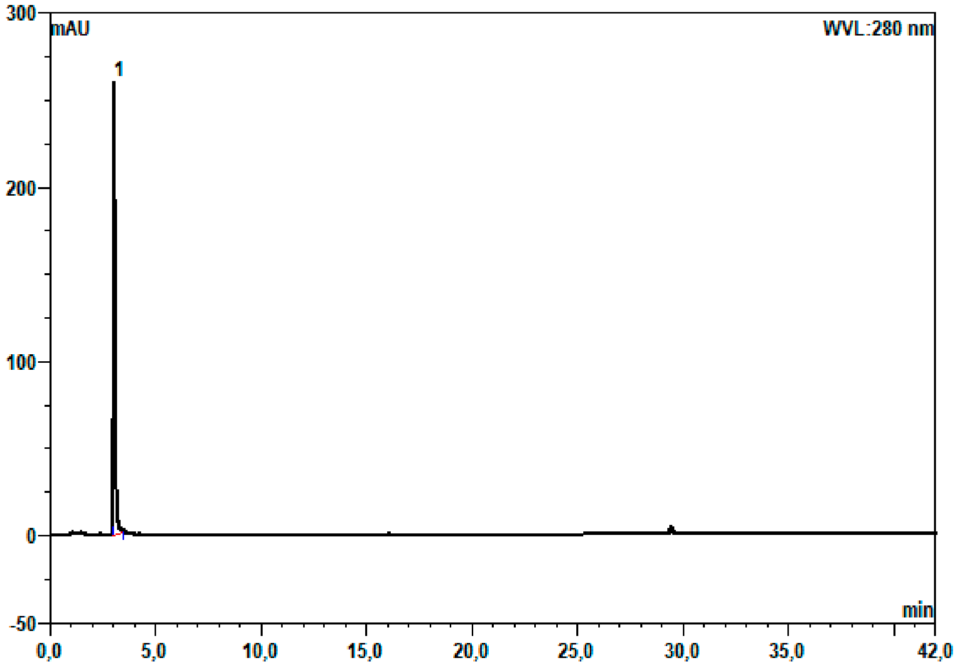

Figure S11. UHPLC-DAD chromatogram of the phenolic compounds recorded at 280 nm in inclusion complex of gallic acid with HP-β-CD. Peak labels: 1 - gallic acid.

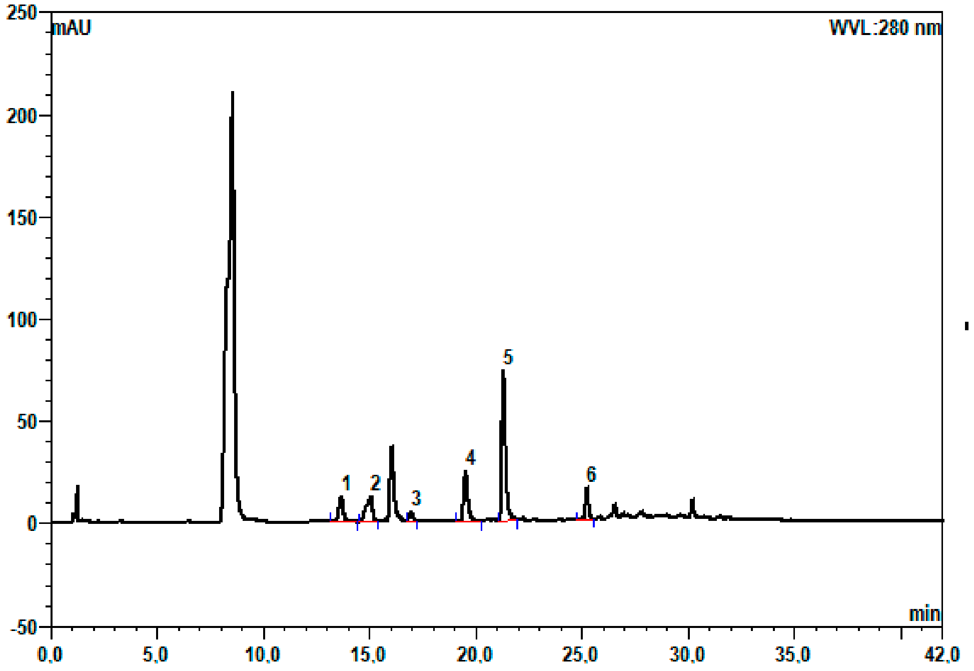

Figure S12. UHPLC-DAD chromatogram of the phenolic compounds recorded at 280 nm in inclusion complex of ACTICOA with β-CD obtained as a result of reactive extraction sample. Peak labels: 1 - caffeic acid aspartate, 2 - (+)–catechin, 3 - *p*-coumaric acid aspartate, 4- procyanidin B2, 5 - (-)–epicatechin, 6 - procyanidin C1.

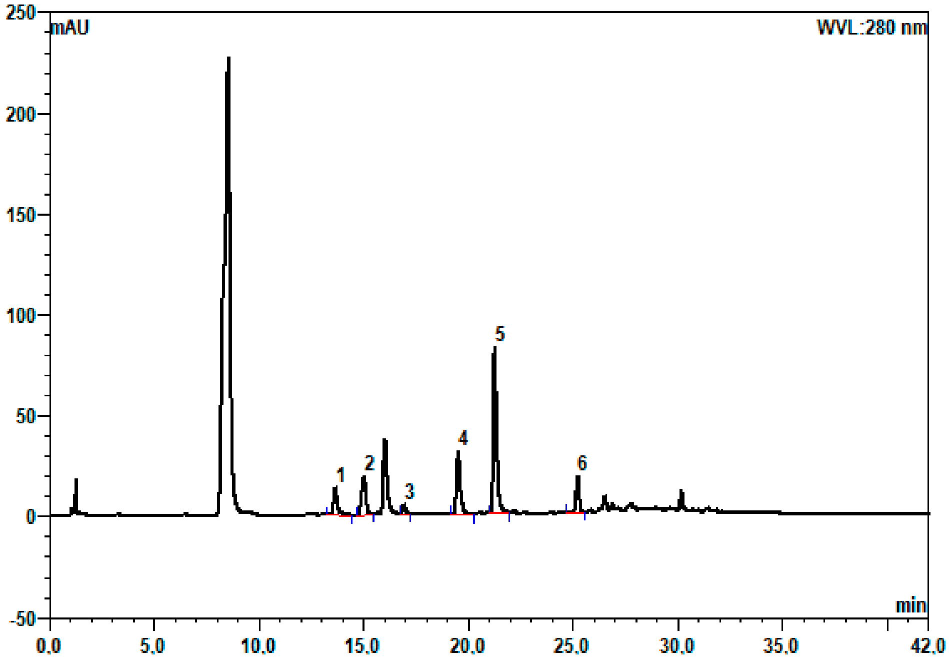

Figure S13. UHPLC-DAD chromatogram of the phenolic compounds recorded at 280 nm in inclusion complex of ACTICOA with HP-β-CD obtained as a result of reactive extraction sample. Peak labels: 1 - caffeic acid aspartate, 2 - (+)–catechin, 3 - *p*-coumaric acid aspartate, 4- procyanidin B2, 5 - (-)–epicatechin, 6 - procyanidin C1.

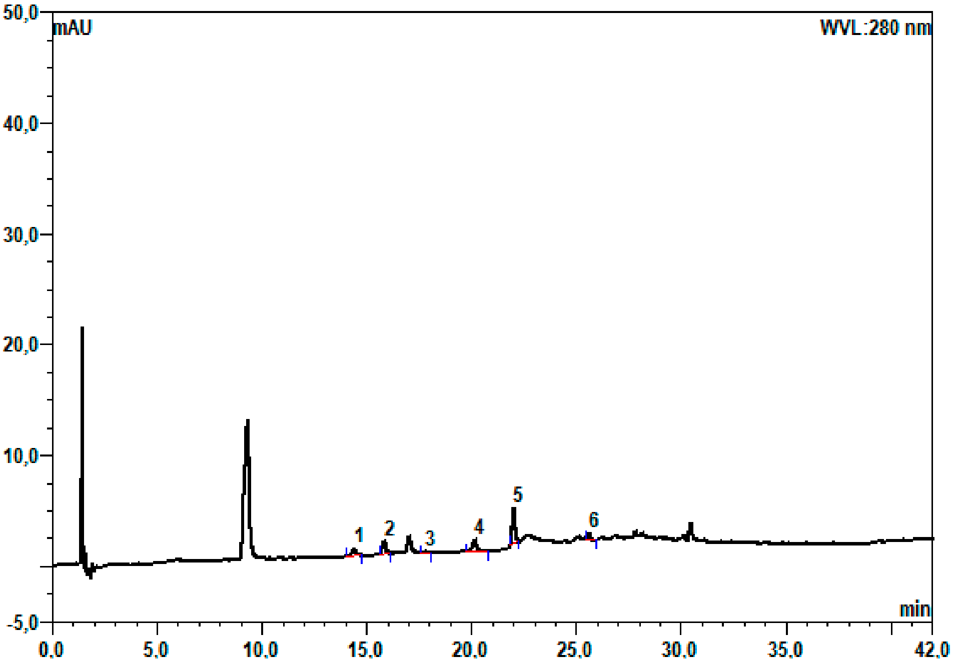

Figure S14. UHPLC-DAD chromatogram of the phenolic compounds recorded at 280 nm in alginate-chitosan capsules with EACTICOA obtained according to method 1. Peak labels: 1 - caffeic acid aspartate, 2 - (+)–catechin, 3 - *p*-coumaric acid aspartate, 4- procyanidin B2, 5 - (-)–epicatechin, 6 - procyanidin C1.

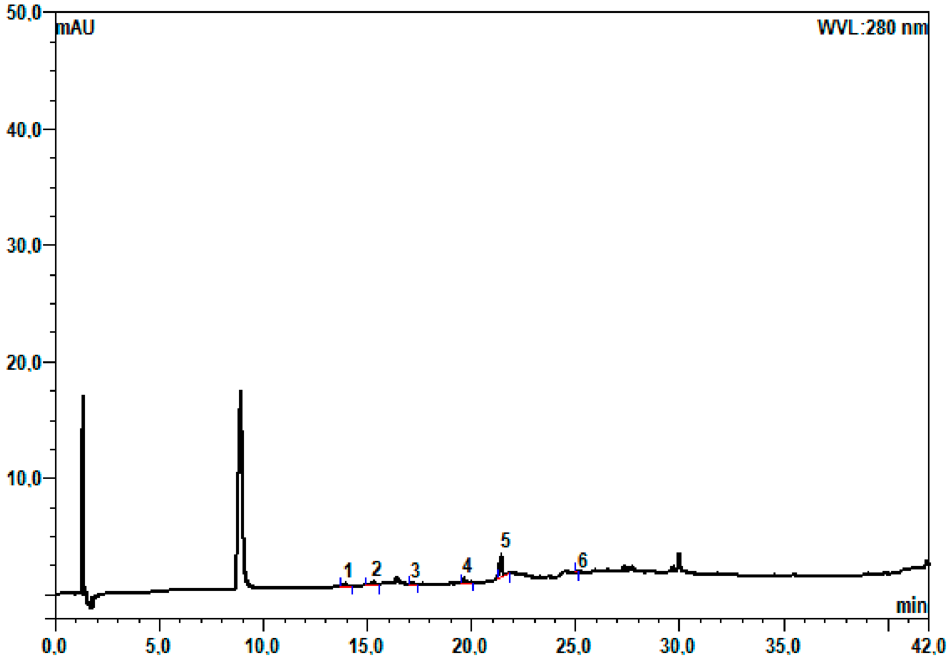

Figure S15. UHPLC-DAD chromatogram of the phenolic compounds recorded at 280 nm in alginate-chitosan capsules with EACTICOA obtained according to method 2. Peak labels: 1 - caffeic acid aspartate, 2 - (+)–catechin, 3 - *p*-coumaric acid aspartate, 4- procyanidin B2, 5 - (-)–epicatechin, 6 - procyanidin C1.

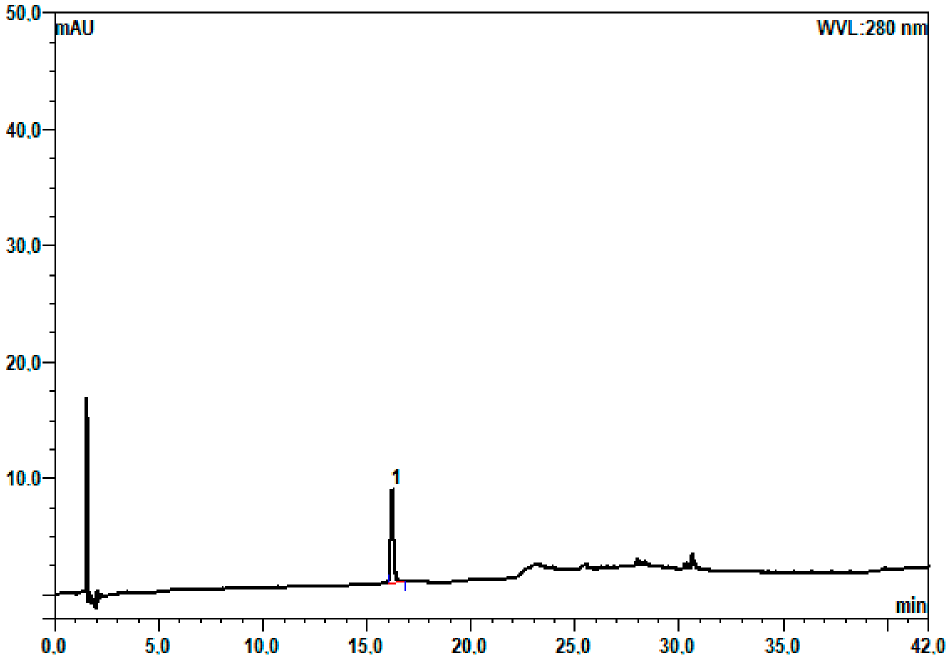

Figure S16. UHPLC-DAD chromatogram of the phenolic compounds recorded at 280 nm in i alginate-chitosan capsules with (+)-catechin obtained according to method 1. Peak labels: 1 - (+)-catechin.

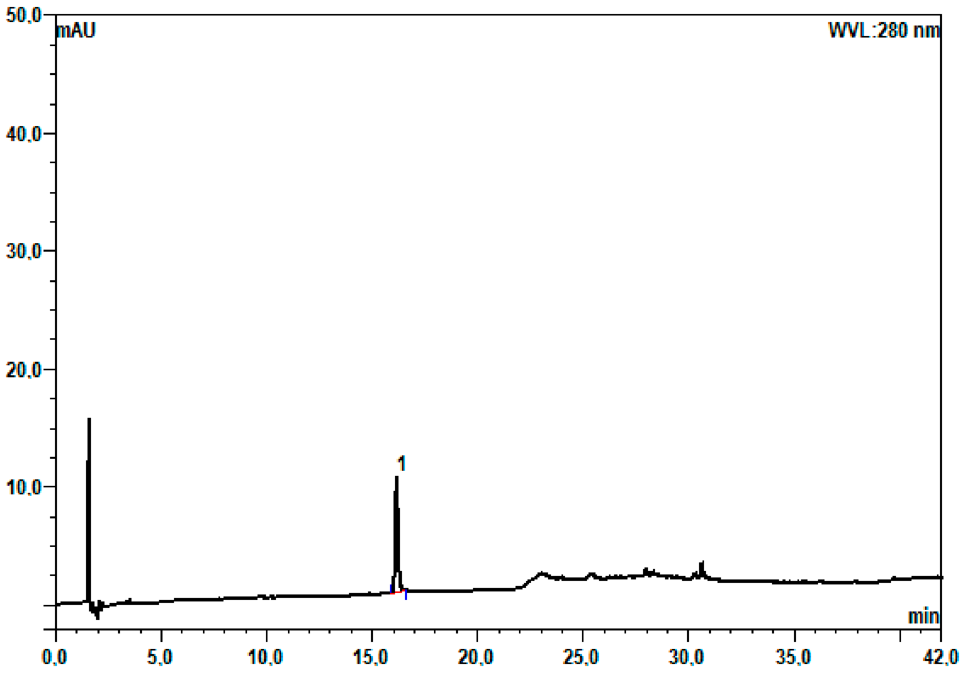

Figure S17. UHPLC-DAD chromatogram of the phenolic compounds recorded at 280 nm in i alginate-chitosan capsules with (+)-catechin obtained according to method 2. Peak labels: 1 - (+)-catechin.

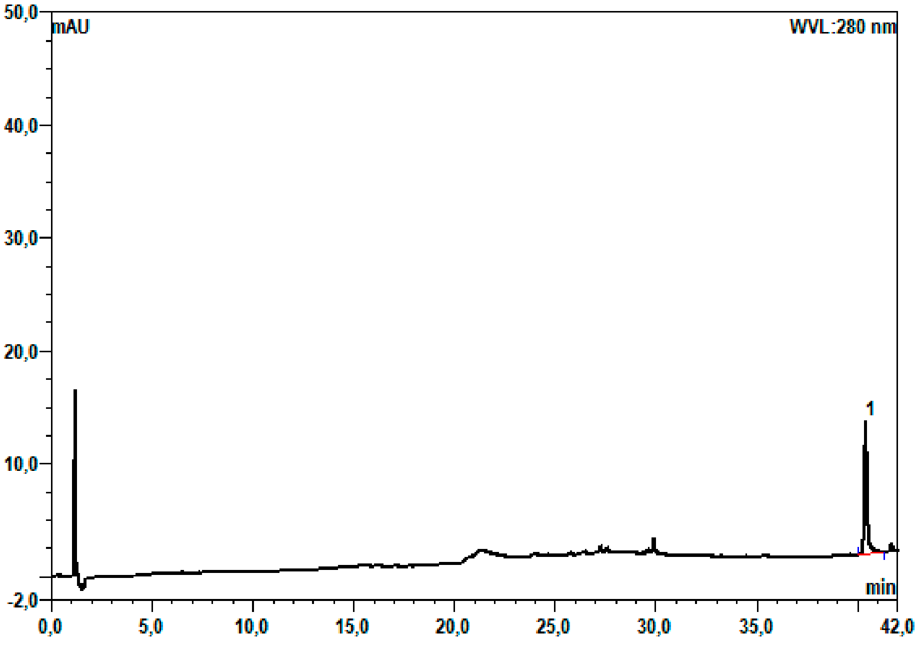

Figure S18. UHPLC-DAD chromatogram of the phenolic compounds recorded at 280 nm in i alginate-chitosan capsules with quercetin obtained according to method 1. Peak labels: 1 - quercetin.

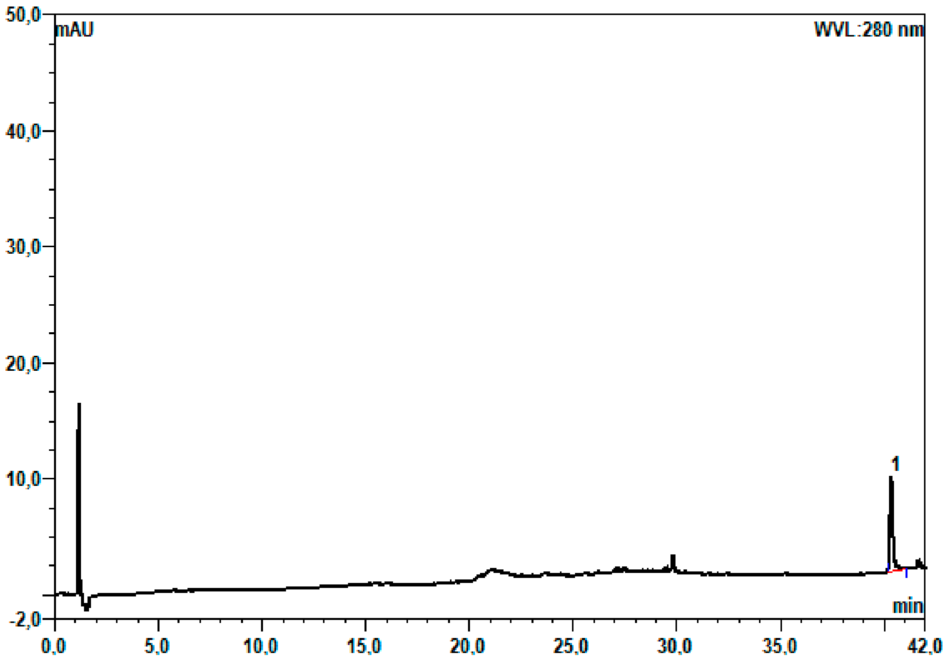

Figure S19. UHPLC-DAD chromatogram of the phenolic compounds recorded at 280 nm in i alginate-chitosan capsules with quercetin obtained according to method 2. Peak labels: 1 - quercetin.

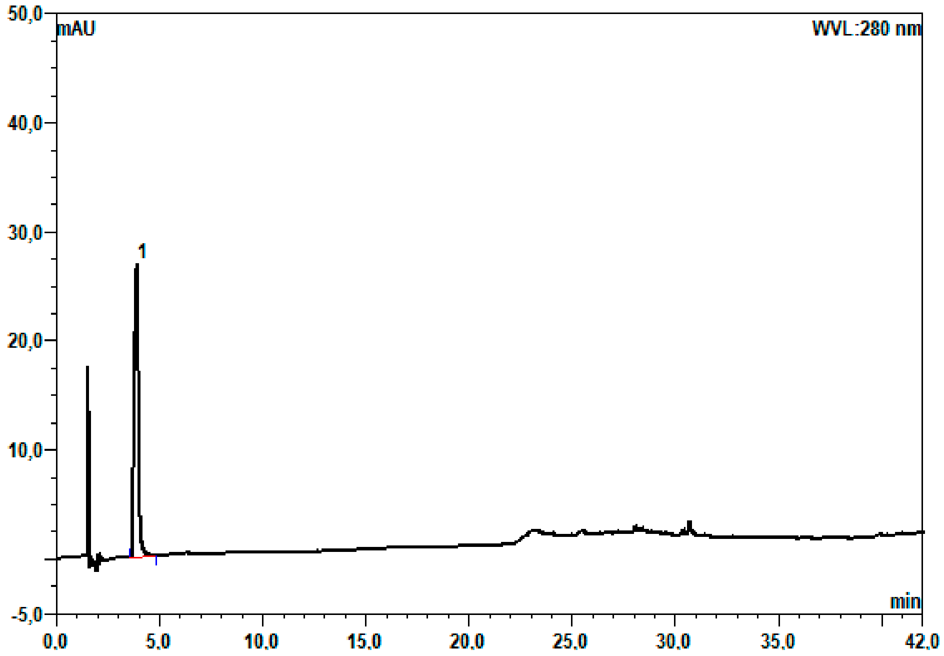

Figure S20. UHPLC-DAD chromatogram of the phenolic compounds recorded at 280 nm in i alginate-chitosan capsules with gallic acid obtained according to method 1. Peak labels: 1 - gallic acid.

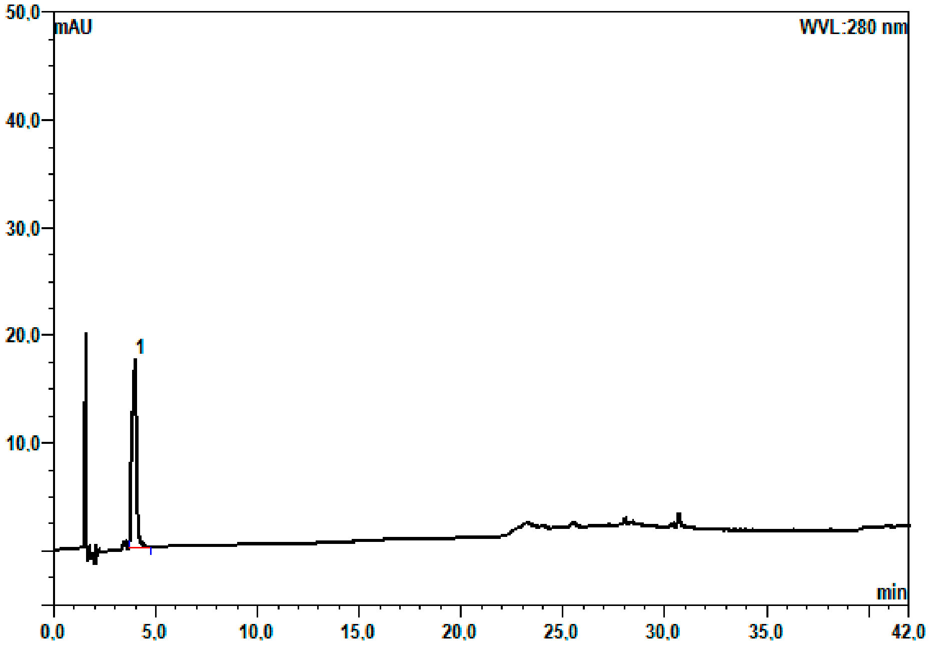

Figure S21. UHPLC-DAD chromatogram of the phenolic compounds recorded at 280 nm in i alginate-chitosan capsules with gallic acid obtained according to method 2. Peak labels: 1 - gallic acid.

Table S1. UHPLC-DAD-ESI-MS/MS identification of phenolic compounds in ACTICOA, EACTICOA, pure phenolic compounds and their encapsulates.

Peak No.	R_t (min)	Phenolic compound	λ_{max} (nm)	MS $[M-H]^-$ (m/z)	Fragmentation ions MS^2 (m/z)
1	2.89	Gallic acid	270.3	169.01	125.02, 97.03
2	6.18	Protocatechuic acid	259.3, 290.8	153.02	109.03
3	13.45	Caffeic acid aspartate	297.1, 320.0	294.06	179.03
4	14.91	(+)-Catechin	201.0, 278.6	289.07	245.08, 205.05, 179.03, 125.02
5	16.61	*p*-Coumaric acid aspartate	307.3	278.07	235.09, 163.04
6	18.20	Procyanidin B2	199.3, 278.9	577.13	425, 407, 451, 289, 125, 559
7	21.61	(−)-Epicatechin	200.9, 278.3	289.07	245.08, 205.05, 179.03, 125.02
8	25.56	Procyanidin C1	199.3, 278.9	865.20	713.15, 575.12, 407.04, 287.06
